# Comparative proteomics of exosomes secreted by tumoral Jurkat T cells and normal human T cell blasts unravels a potential tumorigenic role for valosin-containing protein

**DOI:** 10.18632/oncotarget.8678

**Published:** 2016-04-11

**Authors:** Alberto Bosque, Lisa Dietz, Ana Gallego-Lleyda, Manuel Sanclemente, María Iturralde, Javier Naval, María Angeles Alava, Luis Martínez-Lostao, Hermann-Josef Thierse, Alberto Anel

**Affiliations:** ^1^ Departamento de Bioquímica y Biología Molecular y Celular, Facultad de Ciencias, Universidad de Zaragoza, Instituto de Investigación Sanitaria de Aragón (IIS-Aragón), Zaragoza, Spain; ^2^ Research Group for Immunology & Proteomics, Department of Dermatology, University Medical Center Mannheim, Ruprechts-Karls-University, Heidelberg, Germany; ^3^ Instituto de Nanociencia de Aragón (INA), Zaragoza, Spain; ^4^ Division of Microbiology and Immunology, Department of Pathology, University of Utah School of Medicine, Salt Lake City, UT, USA; ^5^ Servicio de Inmunología, Hospital Clínico Universitario Lozano Blesa, Zaragoza, Spain

**Keywords:** leukemia, T cells, exosomes, apoptosis, proteomics

## Abstract

We have previously characterized that FasL and Apo2L/TRAIL are stored in their bioactive form inside human T cell blasts in intraluminal vesicles present in multivesicular bodies. These vesicles are rapidly released to the supernatant in the form of exosomes upon re-activation of T cells. In this study we have compared for the first time proteomics of exosomes produced by normal human T cell blasts with those produced by tumoral Jurkat cells, with the objective of identify proteins associated with tumoral exosomes that could have a previously unrecognized role in malignancy. We have identified 359 and 418 proteins in exosomes from T cell blasts and Jurkat cells, respectively. Interestingly, only 145 (around a 40%) are common. The major proteins in both cases are actin and tubulin isoforms and the common interaction nodes correspond to these cytoskeleton and related proteins, as well as to ribosomal and mRNA granule proteins. We detected 14 membrane proteins that were especially enriched in exosomes from Jurkat cells as compared with T cell blasts. The most abundant of these proteins was valosin-containing protein (VCP), a membrane ATPase involved in ER homeostasis and ubiquitination. In this work, we also show that leukemic cells are more sensitive to cell death induced by the VCP inhibitor DBeQ than normal T cells. Furthermore, VCP inhibition prevents functional exosome secretion only in Jurkat cells, but not in T cell blasts. These results suggest VCP targeting as a new selective pathway to exploit in cancer treatment to prevent tumoral exosome secretion.

## INTRODUCTION

Multivesicular bodies are compartments inside cells different for endosomes and lysosomes. In these compartments there are intralumenal vesicles, which once secreted to the extracellular medium, are termed exosomes. These exosomes have a particular lipid and protein composition, and a size between 40 to 100 nm. These structures were described for the first time during maturation of erythrocytes [1–4]. In 1975 these structures were isolated in calf fetal serum [5]. Thereafter, exosomes have been described to be released in physiological conditions by different cell types like melanocytes [6], T lymphocytes [7, 8], B lymphocytes [9], dendritic cells [10, 11], platelets [12], mast cells [13], trophoblasts [14], and intestinal, prostate and intraocular epithelial cells [15–17]. It has been also described that exosomes are present in colon mucosa [18], in lactating mammary glands [19], human urine [20] and human bronco alveolar fluid [21]. More recent reviews summarize all the biological fluids and cell types where exosomes have been described [22–24].

Our group characterized previously that FasL and Apo2L/TRAIL are stored inside human T cell blasts in multivesicular bodies [25], and that they were rapidly released to the supernatant in their bioactive form associated with exosomes upon re-activation [8, 25], a paradigm that has been confirmed by other groups [24, 26, 27]. This membrane-bound secretion of death ligands allowed these molecules to conserve their pro-apoptotic potential, and participate in the down-modulation of T cell-mediated immune responses. The physiological importance of this mechanism has been demonstrated in mice knockout for the Wiskott-Aldrich syndrome (WAS) protein. WAS deficiency is primarily characterized as an immunodeficiency, that progresses to autoimmunity. In these mice, autoimmunity manifestations are associated with the inability of T cells to secrete functional FasL associated with exosomes [28]. Furthermore, our group characterized a similar situation in CTL clones from Chediak-Higashi patients. These patients suffer also from autoimmunity associated with problems in granule secretion [29]. In addition, a low amount of FasL and/or Apo2L/TRAIL-carrying exosomes has been detected in synovial fluids from rheumatoid arthritis patients [30]. This observation led to the generation of liposomes decorated with Apo2L/TRAIL and their use as a successful treatment in a rabbit model of rheumatoid arthritis [31]. Recently, a similar immunoregulatory role has been described for exosomes produced by activated humans NK cells [32]. Moreover, FasL secretion on the surface of exosomes has been also described in the case of trophoblasts, where it accomplish an important function in the attenuation of the immune response against the fetus, preventing spontaneous abortion [14, 33].

On the other hand, exosome secretion has been also described in different types of tumoral cells [34–36]; see also the Exocarta and Vesiclepedia websites http://exocarta.org/index.html; http://www.microvesicles.org). In some cases, the secretion of exosome-associated FasL by tumoral cells has been correlated with tumor counterattack mechanisms to escape immunosurveillance by T cells [35, 37–42].

Proteomic analysis of exosomes has been done in B cells [43], dendritic cells [44], intestinal epithelial cells [15], in exosomes isolated from malignant pleural effusion [45], human urine [20] and many other cell types and biological fluids, a compendium of which is available at the recent Exocarta and Vesiclepedia websites [46, 47]. More recently, the presence of microRNA in exosomes has drawn attention on this new system of cell to cell communication and regulation of gene expression ([48, 49], see also [50] for a recent review, and the adapted Exocarta and Vesiclepedia websites).

In spite of all the extensive and expanding work performed on exosome characterization, few systematic studies are available comparing proteomics of exosomes produced by tumoral cells with their healthy cell counterpart. Taking into account that exosome secretion is a recognized mechanism of cell-to-cell communication, it would be of interest to make this comparison in order to identify proteins that could perform a pro-transformation task, or, on the contrary, regulatory proteins that are associated with exosomes from healthy cells that are lost in exosomes from tumoral cells.

In this work, we performed a proteomic analysis of exosomes present in human T cell blasts, and we compared with those of the tumoral cell line Jurkat. Our analysis revealed differential protein expression between both types of exosomes. We further characterized the selective enrichment of VCP only in exosomes of tumoral origin. Of interest, Jurkat tumoral cells are more sensitive to cell death induced by the VCP inhibitor DBeQ than normal T cells. Furthermore, VCP inhibition preventsfunctionalexosome secretion only in Jurkat cells, but not in T cell blasts. These results suggest targeting of VCP as a new candidate for anti-tumoral agents.

## RESULTS

### Exosome detection by electron microscopy

**Figure 1:**
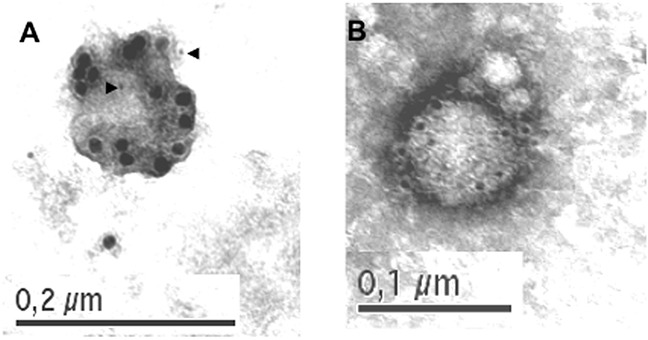
Localization of FasL and Apo2L/TRAIL in exosomes derived from human T cell blasts Human T cell blasts were stimulated with PHA for 5 minutes, PHA washed out, and supernatants collected 1 hour later **A.** or they were stimulated with immobilized anti-CD59 mAb VJ1/12.2 for 3h and supernatants collected **B.**. Exosomes present in supernatants were placed on bacitracin-treated grids as indicated in Material and Methods and observed by TEM. In both cases, FasL (15 nm dots) and APO2L/TRAIL (5 nm dots, marked with arrows in A) were located using, respectively, N20 rabbit pAb and 5C2 mouse mAb, plus secondary anti-rabbit and anti-mouse IgGs.

Previous studies of our group demonstrated by immunoblot and functional assays the secretion of FasL and Apo2L/TRAIL associated with a particulate, ultracentrifugable fraction, during activation-induced cell death of human T cell blasts and tumoral Jurkat cells. We have also showed by immunofluorescence and immunoelectron microscopy (IEM) that FasL and Apo2L/TRAIL were stored inside cytoplasmic multivesicular bodies, associated preferentially with intraluminal vesicle membranes [8, 25]. In this work, for the first time, we show an IEM view of isolated exosomes secreted from T cell blasts pulsed 5 minutes with PHA (Figure 1A) or stimulated during 3h with immobilized anti-CD59 mAb (Figure 1B). Exosomes secreted from PHA-stimulated T cell blasts contained both FasL (15 nm dots), and Apo2L/TRAIL (5 nm dots, marked with arrowheads; Figure 1A). However, in exosomes secreted from anti-CD59 mAb-stimulated T cell blasts, only Apo2L/TRAIL can be detected (Figure 1B). The size of the exosomes are between 100 and 130 nm, in agreement with the unlabeled structures detected previously by scanning electron microscopy [8].

### Lipid composition of exosomes secreted by Jurkat cells or human T-cell blasts

**Figure 2:**
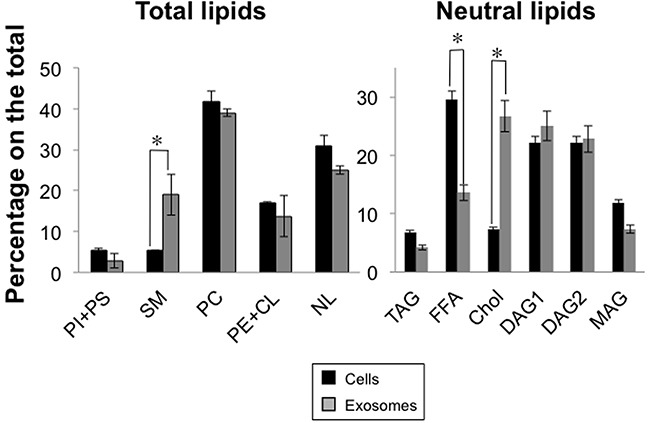
Lipid composition of Jurkat cells and of derived exosomes Cells were first metabolically labeled with [^14^C]-acetate during 72h. Then, cells were stimulated with PHA as indicated in the legend of Figure 1, cells separated from supernatants by centrifugation, and exosomes isolated from the supernatants as indicated in Materials and Methods. Cell or exosome lipids were extracted and subjected to thin-layer chromatography, and radiolabelled bands revealed by autoradiography. Bands were marked in the thin layers, scraped, and radioactivity determined in a b-counter by liquid scintillation. Results are shown as percentage of the total labeling (left graphic) or of the labeling corresponding to neutral lipids (right graphic). PI, phosphatidylinositol; PS, phosphatidylserine; SM, sphingomyelin; PC, phosphatidylcholine; PE, phosphatidylethanolamine; CL, cardiolipin; NL, neutral lipids; TAG, triacylglycerol; FFA, free fatty acids; Chol, cholesterol; DAG, diacylglycerols; MAG, monoacylglycerol. The results are expressed as the mean±SD of two different analysis. *, *P*<0.05.

Lipid composition of Jurkat cells and of exosomes was analyzed as indicated in Materials and Methods. First, we separated the different phospholipid classes from neutral lipids. The main difference between exosome and cell lipids was the enrichment in sphingomyelin (SM) in exosome lipids. We also observed partial reductions in the PI+PS percentage and also in that of neutral lipids, being PC the major lipid in both cases (Figure 2). Afterwards, we separated neutral lipids classes. The main difference in this fraction was the enrichment in cholesterol, with a concomitant reduction in the free fatty acid amount (Figure 2). These differences between exosome and cellular lipids were also observed in the T cell blast model (data not shown). This lipid composition makes exosomes more rigid structures than cell membranes, and defines them as non-fusogenic vesicles. These results are mostly in agreement with previous lipid analysis of exosomes produced from B cells [43].

### Immunoblot detection of specific proteins associated with jurkat and T-cell blasts exosomes

**Figure 3:**
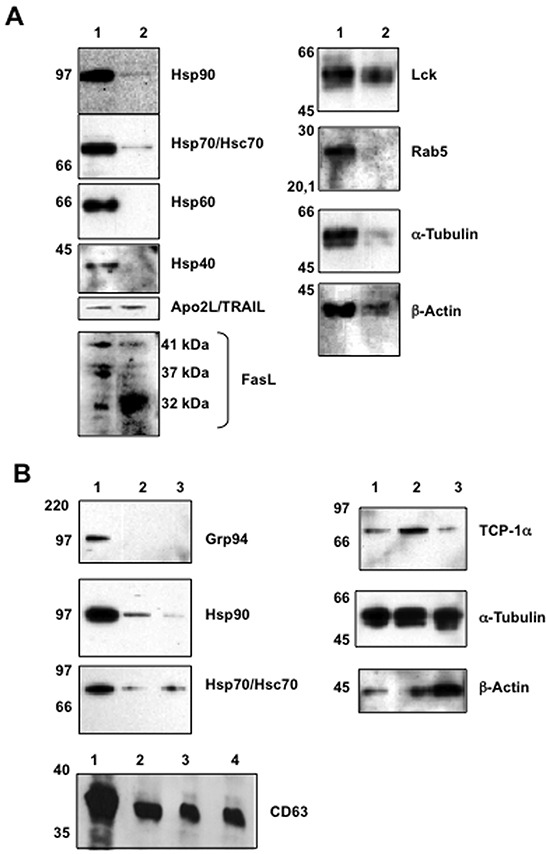
Initial immunoblot analysis of proteins expressed in cells and in exosomes Protein extracts were obtained from cells or from exosomes, proteins separated by electrophoresis in Nu-PAGE Bis-Tris 12% gels, proteins transferred to PVDF membranes and proteins revealed by immunoblot using specific antibodies. In all cases, extracts correspond to 5 μg of total protein. **A.** proteins from whole Jurkat cell extracts (1) or from exosomes secreted by Jurkat cells after pulse-stimulation with PHA (2). **B.** proteins from whole Jurkat cell extracts (1), from exosomes secreted by Jurkat cells after pulse-stimulation with PHA (2) or from exosomes secreted by human T cell blasts pulse-stimulated with PHA (3). In the case of CD63, proteins from non-stimulated Jurkat cells (1), from Jurkat cells pulse-stimulated with PHA (2), from exosomes secreted by Jurkat cells after pulse-stimulation with PHA (3) or from exosomes secreted by human T cell blasts pulse-stimulated with PHA (4). Molecular weights are indicted on the left.

Before beginning the proteomic analysis of exosomes, we decided to characterize the purity of isolated exosomes by immunoblot. One one hand, we were able to detect the expression of the cytosolic chaperones Hsp90 and Hsp70 in exosomes from Jurkat cells. Hsp70 expression was detected by MALDI-TOF analysis in exosomes of dendritic cells [44]. On the other hand, the mitochondrial chaperone Hsp60 and the chaperone Hsp40 were not detected in those exosomes, confirming the specificity in the exosome isolation process (Figure 3A). Furthermore, we detected the presence of the death ligands Apo2L/TRAIL and FasL in these isolated exosomes, confirming previous data ([8, 25] and Figure 1). FasL is synthesized as a 32 kDa polypeptide that is glycosylated during their synthesis. Partial glycosylation generates a doublet at around 37 kDa and full glycosylation generates a mature form of 41 kDa. While all the isoforms are equally represented in cell extracts, exosomes contained predominantly the non-glycosylated polypeptide (Figure 3A).

As previously reported for exosomes of human T cell origin[51], we also detected the protein tyrosine kinase p56^lck^. The microtubule and cytoskeleton proteins α-tubulin and β-actin were also detected in these exosomes, as previously described on exosomes from different cell types. Finally, the vesicular transport protein Rab5 was not detected, indicating again the specificity of protein distribution in the isolated exosomes (Figure 3A).

The exosomes from T cell blasts share protein expression with those from Jurkat cells, compare the corresponding lanes in Figure 3B. Both types of exosomes contain the chaperones Hsp90, Hsp70 and TCP-1α, the tetraspanin CD63, and also α-tubulin and β-actin. In addition, we showed the absence of the ER soluble marker Grp94 in exosomes from both Jurkat cells and T cell blasts, as an additional control of specific protein distribution in exosomes (Figure 3B).

### 2D separation of cell and exosome proteins and MALDI-TOF identification of exosomal proteins

**Figure 4:**
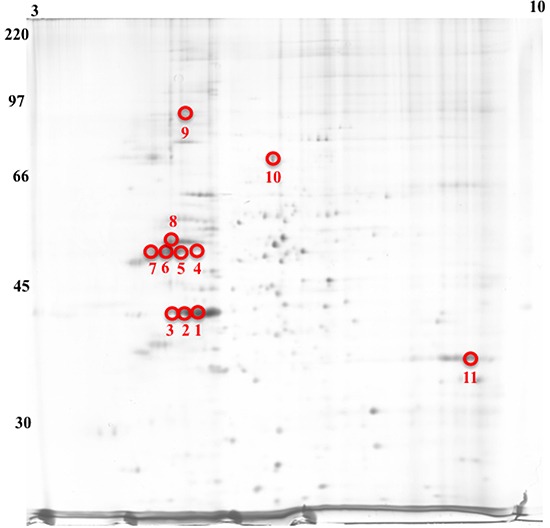
2D separation of proteins in Jurkat exosomes A 2D gel was performed on a sample of 30 μg total protein extracted from Jurkat exosomes and protein spots silver-stained. Isoelectric point is indicated above and molecular weight on the left. The image is representative of at least 5 different gels performed. Numbered spots were identified by MALDI-TOF, see text of Results for details.

As a first approach to the proteomic characterization of exosomes from Jurkat and T cell blasts, we performed 2D electrophoresis separation of proteins obtained from cells or from exosomes. As shown in Supplementary Fig 1, the proteome of Jurkat cells is much more complex than that of their derived exosomes or of the exosomes derived from T cell blasts. The next step was the characterization by MALDI-TOF of proteins obtained from the 2D gels. The identification of several of the major proteins by this technique is indicated in Figure 4, on a 2D gel corresponding to proteins from Jurkat exosomes. The samples marked from 1 to 3 correspond to actin isoforms, and those marked from 4 to 7 correspond to tubulin isoforms. Those are major structural proteins of the cells, which have been identified in exosomes from all cell types studied to date. In addition, we detected 4 major proteins:

- vimentin (sample 8), protein included in the intermediate filaments of class III and implicated in vesicular traffic.
- major vault protein (MVP; sample 9), protein of unknown function, that is indirectly related with vesicular transport between the nucleus and the cytoplasm
- p85Mcm or minichromosome maintenance protein 7 (MCM7; sample 10), nuclear protein implicated in cell cycle control and proliferation, not previously described in association with exosomes
- heterogeneous nuclear riboprotein A2/B1 (HNRPA2B1; sample 11), a protein quantitatively important in the composition of these exosomes. This protein has been recently shown to be involved in the enrichment of specific miRNAs in exosomes[52].

All these proteins were identified in similar analysis made on exosomes from T cell blasts, except p85Mcm/MCM7, that was not detected, indicating an exclusive association with tumoral exosomes.

Additional analysis performed revealed 30 additional proteins in exosomes from Jurkat cells or T cell blasts, finding some interesting specificities in their distribution. Hence, 17 proteins were detected in exosomes from both cell types: annexin-I and VI, Na^+^/K^+^ ATPase, GAPDH, nucleolin, tropomyosin-3, 3 histones and 8 ribosomal proteins; 3 proteins were identified only in exosomes from Jurkat cells; CD3-ζ, signal recognition protein (SRP) and Y-box binding protein-1 (YBX-1); and 10 proteins were only detected in exosomes from normal T cell blasts: CD3-δ, β2 integrin, ADP ribosylation factor-6 interacting protein, HLA-I, β2-microglobulin, CD97, CD5, CD48, interferon-induced transmembrane protein-1 (IFITM-1) and Arp2/3.

All these proteins have been identified previously in proteomic analysis of exosomes, although not all of them in exosomes from T cells or leukemic cells (see the Exocarta webpage). It is interesting to note that with a reduced number of proteins identified, a number of specific locations in exosomes from tumoral or normal T cells, have been already found.

### Proteomic analysis of exosomal proteins by LCMSMS

The LCMSMS technique is a technique much more sensitive than 2D separation of proteins followed by MALDI-TOF identification. For example, in the most complete proteomic study performed to date on T cell exosomes, made in murine T cells, 690 proteins have been identified using this technique [52], while we had identified only 43.

**Figure 5:**
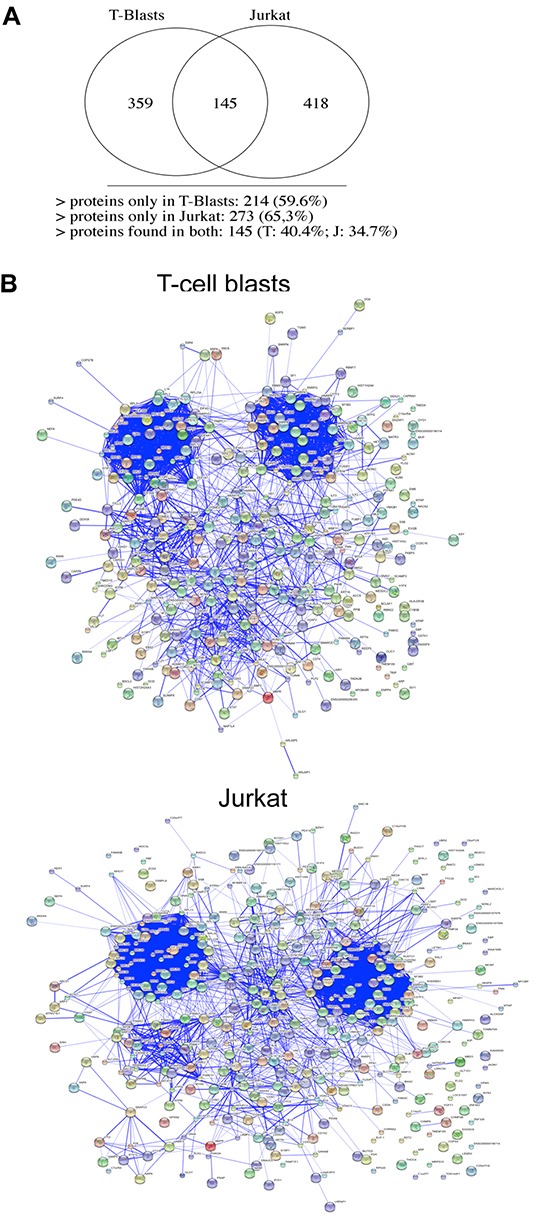
Exosomal proteins detected by LCMSMS 4 different samples obtained from Jurkat cells and samples obtained from 4 different donors were analyzed by LCMSMS. Upper panel, the number of proteins with an statistically significant identification identified in exosomes from Jurkat cells or from human T cell blasts, indicating the number of proteins that were common are represented in a Venn diagram; Lower panels, protein interaction networks obtained using the STRING software between proteins identified in human T cell blast exosomes (middle panel) or in Jurkat cells (lower panel).

Hence, we have performed a complete LCMSMS study, using exosomes from Jurkat cells or from normal T cells blasts, wit the necessary repetitions. We have identified 359 proteins in exosomes from T cell blasts and 418 in exosomes from Jurkat cells, of which only 145 (around a 40%) are common (Figure 5A). The complete list of all proteins identified, separating those that are shared, and those expressed exclusively in exosomes from Jurkat tumoral cells or from normal human T cell blasts, is shown in Supplementary Table 1.

The clustering of these proteins by their interaction profiles using the STRING software shows two defined and major interaction nodes in both T cell blasts and Jurkat T cells. This two major clusters corresponded to ribosomal proteins (cluster at the top left) and to mRNA granule proteins (cluster at the top right; Figure 5B). There is another common cluster of interaction, corresponding to proteins implicated in vesicular traffic. In the case of T cell blasts, there is another cluster of interaction, which is absent in exosomes from Jurkat cells. This cluster corresponds to membrane T cell proteins that stimulate or suppress T cell function, such as Fas/CD95, CD44, CD97, CD5, CD38, CD46, and other interacting proteins. In Jurkat cells, another interacting cluster appears between the two upper ones, corresponding to actin, tubulin, and cytoskeletal and microtubule interacting proteins. Although this cluster is not clearly detected in exosomes from T cell blasts, these proteins are the major ones in these exosomes, so it is not a cluster exclusive of tumoral Jurkat cells.

### Membrane proteins preferentially enriched in Jurkat and T-cell blasts exosomes

It is tempting to speculate that mostly membrane proteins could have a specific function in exosomes, since most of the proteins found inside exosomes are rather cytoplasmic proteins localized there due to the mechanism of exosome generation by inward vesiculation of the external MVB membrane. Hence, we have separated the membrane proteins that are detected by proteomic techniques only in exosomes from Jurkat cells or from T cell blasts. We show in Table 1 that, while in T cell blast exosomes predominate normal “cluster of differentiation “(CD) immune membrane proteins, with their known regulatory functions, those were mostly absent from Jurkat exosomes. These proteins are coincident with those present in the cluster or interaction that we showed specific for T cell blasts in Figure 5B. In addition, components of the antigen presentation machinery, including several HLA proteins, β2-microglobulin and the invariant chain (CD74) are detected exclusively in the membrane of T cell blast exosomes. On the contrary, 14 membrane proteins are detected only in exosomes from Jurkat cells and could have a previously non-described role in malignancy (Table 1). Most of these proteins (VAMPs, VAMP-associated proteins, dynein, syntaxin 12, lamp1) are related with vesicular traffic processes. However, the most abundant of these proteins was VCP.

**Table 1:** Membrane proteins differentially detected by MS-based methods in exosomes from T cell blasts or Jurkat cells

Only in T cell blasts exosomes (25)	Only in Jurkat cell exosomes (14)
Syntaxin 7	CD3ζ*
Syntaxin 4	Myobrevin (VAMP5)
Integrin β2	Cellubrevin (VAMP3)
HLA-I-B & C	VAMP-associated protein B (VAPB)
2-microglobulin	VAMP-associated protein A (VAPA)
CD74 (invariant chain)	ICAM2
CD3δ	ATP6V1EI
CD5	Dynein
CD46	Lamin B receptor
CD2	Ryanodine receptor
CD63**	Syntaxin 12
CD48	Lamp1
CD58	VCP
CD44	KDEL retention receptor 2
CD38	
CD6	
CD97	
IFITM1	
IFITM3	
Fas (CD95)	
ICAM3	
LAIR1	
SLAMF6	
LFA-1	

* (only detected by MALDI-TOF)

** (detected by immunoblot in Jurkat cell exosomes)

### VCP experiments

**Figure 6:**
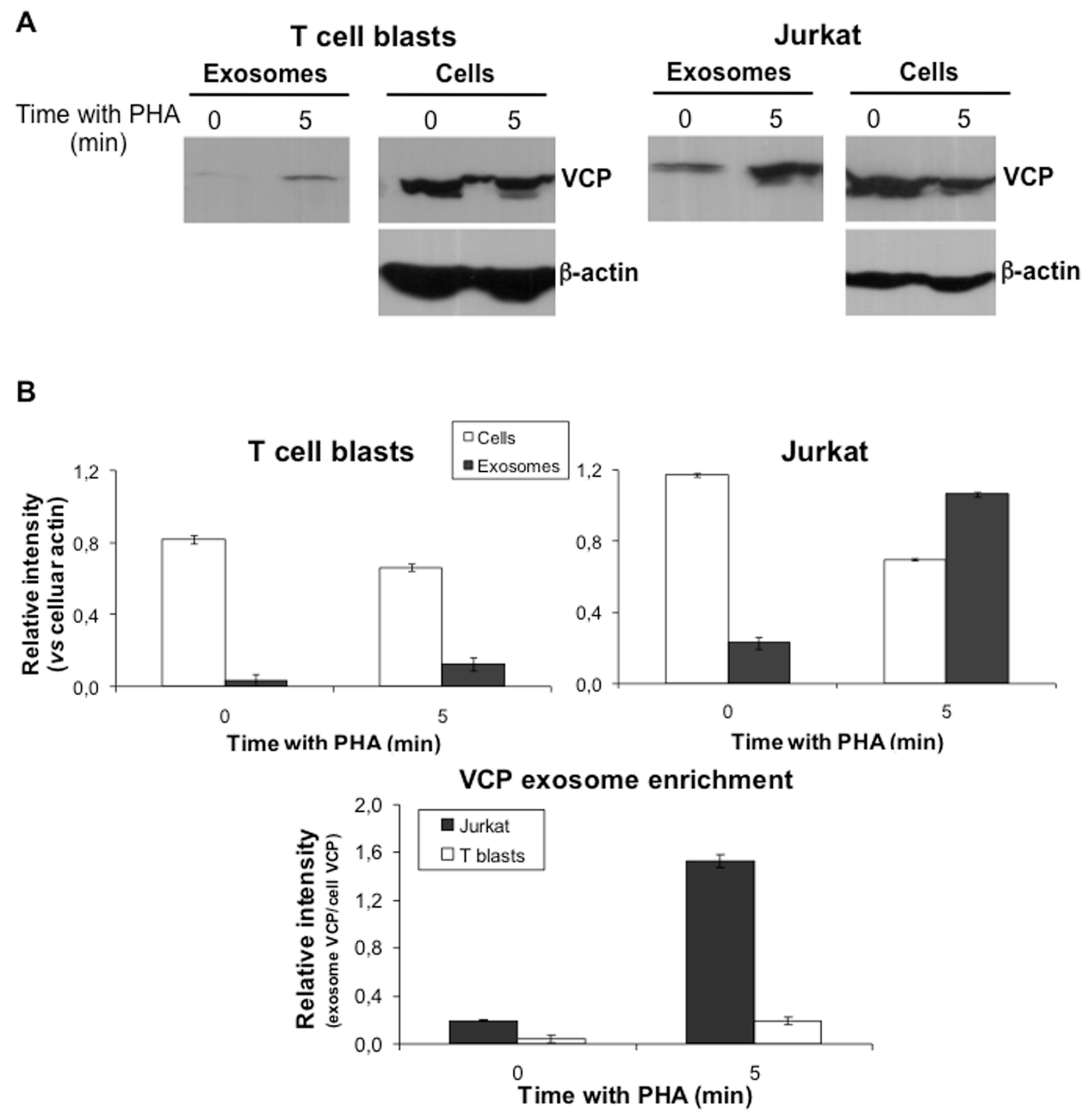
Analysis of VCP expression in cells and exosomes **A.** Levels of VCP protein both in cell extracts and in exosomes were analyzed by Wetern blot. Both T cell blastas and Jurkat cells were unstimulated or stimulated with PHA for 5 min. Then, PHA was removed and cells were suspenden in fresh medium. Finally, supernatants were collected 1h later and proteins extracted. Cell and exosome proteins were separated by electrophoresis and VCP immunoblotted with an specific antibody. The levels of β-actin were also determined in the same gels as a control of protein loading. **B.** Upper graphs show the relative intensity of the VCP bands as a ratio with cellular β-actin expression, analyzed using the ImageJ software. Lower graph shows the VCP enrichment in exosomes, expressed as the ratio between exosome and celular VCP in each experimental condition. The results are expressed as the mean±SD of two different experiments.

p97/VCP is a membrane ATPase involved in ER homeostasis and ubiquitination [53]. It has been associated with tumoral transformation, having prognostic value, at least in gastric carcinoma [54]. First of all, we confirmed this specific distribution of VCP in Jurkat exosomes by immunoblot. As shown in Figure 6, VCP is an abundant protein both in T cell blasts and in Jurkat cells at the basal stage. The protein is not detected in exosomes from T cell blasts culture supernatants, but it could be already detected in exosomes from Jurkat cell supernatants at the basal stage. When T cell blasts or Jurkat cells were pre-stimulated with PHA for 5 min, PHA washed, cells resuspended in fresh medium, and supernatants collected 1h later, as described above in our exosome proteomic analysis, VCP release associated with exosomes was much higher in the case of Jurkat cells than in the case of T cell blasts. In fact, the amount of VCP secreted from Jurkat cells in 1h, expressed as the ratio with cellular β-actin, is higher that the amount still present inside the cells. In the case of normal T cell blasts, although a small amount of VCP could be detected in the supernatant after 1h, most VCP is still present inside the cells (Figure 6B). In fact, VCP was enriched in Jurkat exosomes at an 8-fold higher rate than in T cell blasts exosomes after activation, as shown in the lower panel of Figure 6B.

**Figure 7:**
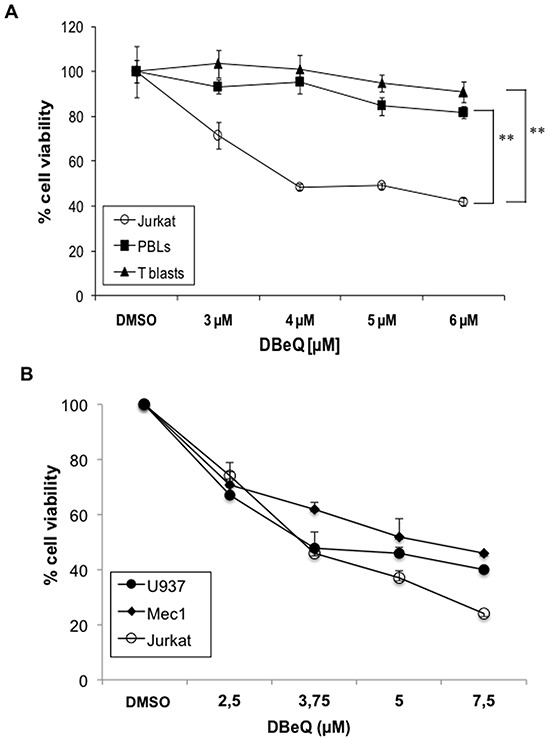
Effect of the VCP inhibitor DBeQ on leukemic and normal lymphocytes Dose-response assays using the indicated doses of DBeQ were performed on freshly isolated PBMC, derived T cell blasts and Jurkat cells **A.**, or on leukemic U937, Mec1 and Jurkat cells **B.**. The decrease in cell viability was measured using the MTT assay after 24h of incubation. The results are expresses as the mean±SD of at least two different experiments. **, *P* < 0.01.

We further tested whether the enrichment of VCP in exosomes from tumoral cells have a physiological role. To that end, we used a recently developed reversible VCP inhibitor, termed DBeQ. This diamine was characterized by high-throughput screening between more than 200,000 compounds as the most potent and specific for p97/VCP inhibition [62]. As shown in Figure 7A, DBeQ was toxic for tumoral Jurkat cells, but not for freshly isolated PBMC or derived T cell blasts at doses up to 6 μM. This result indicates that VCP activity is needed for tumoral cell survival, while normal human T cells are not so dependent on this ATPase for survival. We extended these results obtained in Jurkat cells to other human leukemias, such as the promonocytic leukemia U937 and the chronic lymphocytic leukemia B cell line Mec1, which showed similar IC_50_ as the acute T cell leukemia Jurkat (Figure 7B).

**Figure 8:**
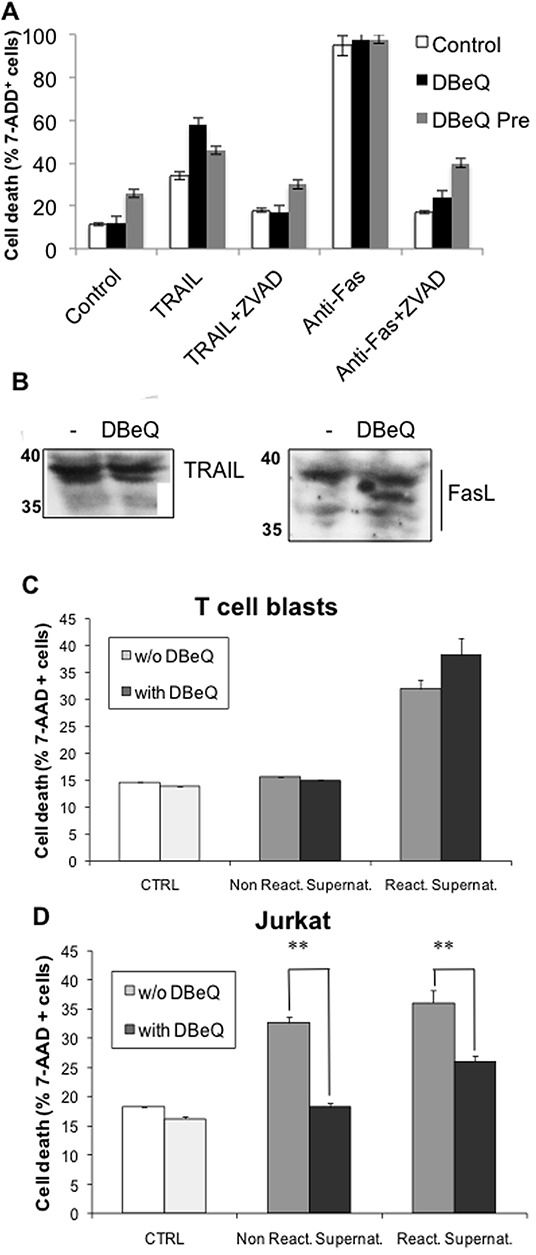
Effect of the VCP inhibitor DBeQ on exosomes release from T cell blasts or from tumoral Jurkat cells **A.** Jurkat cells were either left untreated (control) or they were treated overnight with 1 μg/ml of soluble TRAIL or with 50 ng/ml of the anti-Fas mAb CH11, in the presence or absence of 30 μM of the caspase inhibitor Z-VAD-fmk, as indicated (white bars). The possible effect of DBeQ was tested using two incubation protocols. In the first one (black bars), 3 μM DBeQ was present during the overnight assay, and in the second (grey bars), cells were pre-incubated with 3 μM for 16h before the incubation with anti-Fas of with TRAIL and the assay performed in the absence of DBeQ. Cell death was determined by flow cytometry using 7-amino-actinomycin D (7-AAD) staining. The results are expressed as the mean±SD of at least two different experiments. **B.** Jurkat cells were incubated in the presence or absence, as indicated, of 3 μM DBeQ for 16h, cell extracts were obtained, and the expression of TRAIL or FasL was determined by immunoblot. **C, D.** T cell blasts and Jurkat cells were cultured in the presence or in the absence of 3 μM DBeQ for 16h. Then, DBeQ was removed and cells were stimulated with 10 ng/ml phorbol myristate-acetate (PMA) plus 600 nM ionomycin during 2h. After that, cell supernatants were collected by centrifugation, and their cytotoxicity was tested overnight against non-activated Jurkat cells. Finally, cell death was determined by flow cytometry using 7-amino-actinomycin D (7-AAD) staining. The results are expressed as the mean±SD of at least two different experiments. **, *P* < 0.01. **C.** Cytotoxic effect of supernatants from T cell blasts. **D.** Cytotoxic effect of supernatants from Jurkat cells.

To investigate the effect of DBeQ on exosome release, we used a bioassay previously optimized by our group [55, 56]. Briefly, supernatants of T cell blasts or Jurkat cells stimulated with PMA plus ionomycin are tested against non-stimulated Jurkat cells. In our previous studies, we have shown that cytotoxicity on Jurkat cells of these supernatants is mainly due to FasL and Apo2L/TRAIL secretion associated with exosomes [8, 56, 57], being thus a functional test of exosome secretion. Before performing the bioassays to test exosome secretion in the presence of DBeQ, we demonstrated that DBeQ does not inhibit anti-Fas mAb or recombinant TRAIL-induced apoptosis on Jurkat cells, while the general caspase inhibitor Z-VAD-fmk does inhibit death receptor-induced apoptosis, as previously reported (Figure 8A). The absence of DBeQ effect on Fas- or TRAIL receptor-induced apoptosis was observed either if 3 μM of the VCP inhibitor was present during the overnight assay or if cells were pre-incubated during 16h with DBeQ and then washed out before the assay. As an additional control, we show that incubation during 16h with this concentration of DBeQ does not decrease FasL or TRAIL expression in Jurkat cells (Figure 8B). As shown in Figure 8C, supernatants from non-stimulated T cell blasts, pre-incubated with or without DBeQ, are not cytotoxic against Jurkat cells. In addition, supernatants from re-activated T cell blasts in the presence or absence of DBeQ were equally cytotoxic against Jurkat cells. In the case of supernatants from non-stimulated Jurkat cells, we could detect some cytotoxicity, that is increased after PMA + ionomycin stimulation. In both cases, preincubation with DBeQ inhibited significantly the secretion of cytotoxic exosomes from Jurkat cells (Figure 8D). Our results indicate that VCP is needed for the secretion of exosomes from tumoral Jurkat cells, but not from normal human T cell blasts. These results also point to a higher basal level of functional exosome generation in the case of tumoral Jurkat cells than in the case of normal human T cell blasts.

## DISCUSSION

To our knowledge, this is the first systematic and proteomic study of exosomes secreted by human leukemic cells and their healthy counterparts.

In the present study we have confirmed our previous data on FasL and Apo2L/TRAIL functional secretion in association with exosomes [8, 25], by additionally showing the presence of FasL and/or Apo2L/TRAIL in isolated exosomes by IEM and by immunoblot. This has been recognized as a mechanism of immune down-modulation [27, 28], also in the prevention of abortions [58], and the release of FasL-carrying exosomes by tumor cells recognized as a mechanism of tumor counter-attack against immune surveillance [39–41]. However, it is remarkable that neither FasL nor Apo2L/TRAIL have been detected by proteomic analysis using MS-detection based methods. This could be due to the glycosylation nature of these membrane proteins, which could decrease their solubilization and/or to their trypsin processing. It is remarkable that in a thorough proteomic study in which two different intracellular granule populations are detected in activated human T cells, characterized by the presence of FasL in one of them and of granzyme B in the other population, FasL was also not detected by ME-based proteomics even in the granules that were characterized by their presence [59]. Also, perforin and granzyme B have not been detected in exosomes, but granzyme A has been detected exclusively in exosomes from human T cell blasts, but not in exosomes from Jurkat cells, as expected from the presence of cytotoxic CD8^+^ T cells in the blast population. The amount of granzyme A is normally higher than that of granzyme B in cytotoxic T lymphocytes, and its detection in exosomes is in agreement with the previous observation on the presence of granzymes not only in simple cytoplasmic granules, but also in MVB inside CTL, being secreted afterwards associated with exosomes which also express the TCR/CD3 complex [7]. Indeed, we have detected CD3 components in exosomes from both T cell blasts and Jurkat cells, also in agreement with a previous immunoblot study on exosomes from human T cells [51].

The proteins identified in our study are mainly coincident with the most thorough study on T cell exosomes published to date, although in that case corresponded to exosomes from murine T cells [60]. Major proteins included actin, tubulin, cytoskeleton and microtubule related proteins, and also ribosomal and mRNA granule proteins. Especially interesting is the presence of the tetraspanin CD81, expressed in exosomes from both Jurkat cells and T cell blasts, and that has been recently described to direct the sorting of proteins towards exosomes [60].

The fact that the major proteins in exosome composition are cytoskeletal, ribosome and mRNA granule proteins reflects the mechanism of exosome generation, by inward vesiculation of the limiting multivesicular membrane. This makes that the major exosome proteins are indeed the major cytoplasmic proteins, including cytoskeletal proteins, ribosomal and mRNA granule proteins in cells that are activated and dividing. Hence, we propose that membrane proteins have a higher chance to perform specific functions. This is not an affirmation that holds true for all the soluble proteins inside exosomes, for example, one of the major proteins identified in exosomes from both Jurkat and T cell blasts, HNRPA2B1, has been recently shown to increase the expression of specific miRNAs in exosomes [52]. Good examples of membrane proteins expressed on the surface of exosomes and involved in functional responses are FasL, Apo2L/TRAIL, and also CD81, as commented above. However, these proteins are expressed in exosomes from both Jurkat and human T cell blasts, and the main goal of our study was to identify proteins that were exclusively expressed on exosomes from one type or cell or the other. We found that 24 membrane proteins are especially enriched in exosomes from T cell blasts, and that the reverse situation occurred for 14 proteins enriched in Jurkat cell exosomes. Proteins expressed in exosomes from normal human T cell blasts included HLA proteins and β2-microglobulin, and many CD proteins, such as CD2, CD5, CD46, CD48, CD58, CD44, CD38, CD6, CD97, Fas/CD95, LFA-1 and β2-integrin. All these proteins are related with the physiological function of these immune cells, and are lost in the exosomes of leukemic cells. This result suggests that one differential paradigm of exosomes from tumoral cells is the loss of membrane proteins involved in normal cell function.

Regarding membrane proteins that are enriched in exosomes from Jurkat cells, most of them are related with vesicular traffic, indicating that these processes, including exosome generation and secretion, seem to be more active in tumoral cells. This is demonstrated also by the higher secretion rate of exosomes in tumoral cells at the basal level. The most abundant of these proteins was VCP and, as a first possible application of this study, we have validated the different expression of this protein in both types of exosomes. p97/VCP is a membrane ATPase involved in ER homeostasis and ubiquitination [53], that is associated with tumoral transformation, having prognostic value, at least in gastric carcinoma [54]. It has been shown recently that VCP inhibition by the specific inhibitor DBeQ [61] or also by sorafenib [62] could have therapeutic interest in cancer treatment. We show that Jurkat tumoral cells, and also other leukemic human cells, such as U937 and Mec1, are more sensitive to cell death induced by the VCP inhibitor DBeQ than normal human T cells, suggesting a new selective pathway to exploit in cancer treatment. In addition, we show that VCP inhibition preventsfunctionalexosome secretion only in Jurkat cells, but not in T cell blasts. This suggests that VCP is implicated in the process of exosome generation and/or secretion, but this implication is important only in tumoral Jurkat cells, but not in normal human T cell blasts.

Exosome secretion is common in tumoral cells, and it has a pro-tumorigenic function, at least through suppression of anti-tumoral immune surveillance [41]. However, many other unknown mechanisms that promote tumorigenesis could be associated with exosome secretion by tumor cells, such as the transfer of specific miRNAs [50]. Recent studies indicate the specific association of some proteins such as MET in the case of melanoma, and glypican-1 in the case of pancreatic cancer with exosomes, and their role in promoting the malignant transformation [63, 64]. We have not detected these proteins in our exosomes, since their distribution could be tissue-specific, but the targeting of VCP could decrease exosome generation in several tumor models, offering an interesting new avenue for cancer treatment.

The approach to study membrane proteins has been a first step and other membrane or soluble proteins can be studied in future work. For example, YBX-1 and p85Mcm/MCM7 are also major proteins in the exosomes of Jurkat cells, but are absent from the exosomes of T cell blasts. YBX-1 is a translation repressor of specific mRNAs, is over-expressed in some tumors, and it increases the expression of the multidrug resistance receptor (MDR) in especially aggressive tumors [65]. On the other hand, p85Mcm/MCM7 is a nuclear protein belonging to the minichromosome maintenance family, which oncogenic activity has been recently recognized [66].

## MATERIALS AND METHODS

### Cells and cell culture

The Jurkat T-cell leukemia (ATCC, clone E6.1), U937 histiocytic lymphoma, and the Mec1 chronic lymphocytic leukemia cell line were cultured in RPMI 1640 medium (Gibco, Barcelona, Spain) supplemented with 10% fetal calf serum, Image-glutamine and penicillin/streptomycin (hereafter, complete medium) using standard cell culture procedures.

Human peripheral blood mononuclear cells (PBMC) were obtained from blood of healthy donors by Ficoll-Paque density centrifugation, as indicated elsewhere [67]. T-cell blasts were generated as follows: PBMC (2×106 cells/ml) were stimulated during 1 day with 5 μg/ml phytohemagglutinin (PHA). Afterwards, PHA was washed out, cells suspended in complete medium supplemented with 30 UI/ml interleukin-2 and cultured for 5 days with medium changes every 48 h.

### Exosome purification

After washing twice in RPMI-1640 medium without serum, 250 × 10^6^ Jurkat cells or 500 × 10^6^ of T-cells blasts were suspended in 15 ml of this same medium and re-stimulated by incubation with 50 μg/ml PHA at 37°C for 5 min. Cells were washed three times with RPMI-1640, suspended in 15 ml RPMI-1640 and incubated for 1 hour at 37°C. Supernatants were collected by centrifugation for 5 min at 300 × *g*. Supernatants were clarified by two sequential centrifugations, the first at 1,800 × *g* for 20 min and the second at 10,000 *x g* for 30 min. Exosomes were finally isolated by ultracentrifugation at 110,000 *x g* for 8 hours with a SW40 Ti rotor (Beckmann).

### Immunoelectron transmission microscopy of exosomes

Exosomes were isolated from supernatants of T cell blasts after treatment with PHA as indicated above, or with immobilized anti-CD59 mAb for 3h. Immunogold labeling of exosomes was performed essentially as described in [40]. Briefly, formvar-coated copper grids, wet with bacitracin at 7.5 μg/ml for 2 minutes, were placed on 15 μl aliquots of the exosome preparation for 10 minutes, and then fixed with 3% paraformaldehyde for 5 minutes. Double FasL and Apo2L/TRAIL labeling was performed by using the N20 rabbit pAb (lot D159) at 6 μg/ml and the anti-Apo2L/TRAIL mouse mAb 5C2 at 25 μg/ml plus anti-rabbit IgG and anti-mouse IgG coupled to colloidal gold particles 15 nm and 5 nm in size, respectively, at 1/50 dilution. Finally, grids were negatively stained with uranyl acetate for 1 minute, and examined in a Jeol JEM 1010 transmission electronic microscope (Jeol, Barcelona, Spain) at 80 kV. In the conditions described, the labeling was exclusively associated with exosomes, with no labeling outside membranous structures. In these conditions, no labeling was observed when using the secondary antibodies alone or irrelevant rabbit or mouse IgG.

### Lipid analysis

For analysis and quantification of the lipid composition of cells and exosomes secreted from the cells, we first performed [^14^C]-acetate metabolic labeling of the cells during 72h. After that, cells were stimulated as indicated above for exosome secretion, cells separated from supernatants by centrifugation, and exosomes isolated as indicated above. After that, cell or exosome lipids were extracted with chloroform/methanol (2:1, v/v) and lipids separated by thin-layer chromatography (TLC). Phospholipid separation was made using chloroform/methanol/32% ammonia (65:35:5, v/v; [68]) as eluent. Neutral lipid separation was made using the standard eluent hexane/ethyl ether/acetic acid (70:30:1, v/v; [69]). Radiolabelled lipids in TLC plates were located by autoradiography (Hyperfilm β-max, Amersham) at room temperature for 2 days and radioactivity quantified by liquid scintillation counting of scrapped silicagel. In all TLC analysis, positions of the authentic lipids were identified by running commercial standards of all lipid and phospholipid classes (Sigma, Madrid, Spain) in the same plates, and revealing them with iodine.

### 2-D electrophoresis

5×10^6^ cells were washed with PBS and solubilized in 100 μl lysis buffer (6M Urea, 2M Thiourea, 1% Triton, 40 mM Tris, 60 mM DTT and Complete Mini (EDTA free)) by incubating on ice during 30 minutes and centrifugation 15 minutes at 13,500 rpm. Supernatant was collected and protein concentration was quantified with Coomasie^®^ Plus Protein Assay Reagent following the manufacturer's protocol. Exosomes from 250 × 10^6^ Jurkat or 500 × 10^6^ T-cells blasts were lysed in 500 μl of the same lysis buffer and protein concentration was quantified with Coomasie^®^ Plus Protein Assay Reagent following the manufacturer's protocol.

Analytical loadings of 30 μg of protein were suspended in rehydration buffer (6M Urea, 2M Thiourea, 2% CHAPS, 18 mM DTT, 0,8% IPG Buffer, Bromophenol Blue) to a final volume of 350 μl. Samples were applied to IPG strips 18 cm pH 3-10 by in-gel rehydration, over-night at room temperature. Four-step-focusing (step 1, 1h, 500 V, step and hold; step 2, 1000V, step and hold; step 3, 1h, 8000V, linear gradient; step 4, 8h, 8000V, step and hold) was performed with an IPGphor system (Amersham Biosciences, Freiburg, Germany) for 65 kVh. Strips were equilibrated for 60 min in 50 mM Tris-HCl pH 8,8 buffer containing 6 M urea, 30% glycerol, 2% SDS, 1% DTT and 0,01% bromophenol blue, then treated for 60 min in the same buffer containing 2,5% iodoacetamide instead of DTT. The strips were then transferred onto 10% SDS-PAGE gels (200 × 250 × 1.00 mm) for the 2-D separation (DALT system, 90 V constant voltage, overnight run at 20°C).

Silver staining was performed as described before [70]. Silver stained gels were scanned using a LabScan Imager Scanner (Amersham Biosciences, Freiburg, Germany). The resulting 2-D protein patterns were analyzed, using the programs LabScan 3.00 and Image Master 2-D Elite (Amersham Biosciences, Freiburg, Germany).

### Immunoblot analysis

Extracts with urea were dialyzed in two steps. In the first step, extracts were dialyzed against 4M urea, 137 mM sodium chloride, 20 mM tris, 1 mM EDTA and 1% Triton X-100 during 4 hours at 4°C. In the second step, extract were dialyzed against 137 mM sodium chloride, 20 mM tris, 1 mM EDTA and 1% Triton X-100 over night at 4°C. 5 μg of protein were analyzed by NuPAGE™ 12% Bis-Tris Gels (Invitrogen) and separated proteins transferred to PVDF membranes (Millipore).

To analyze supernatants before and after ultracentrifugation, supernatants were treated with 10% trichloroacetic acid (Roth, Germany) during 15 min on ice. Supernatant were centrifuged during 15 min to 13000g at 4°C, washed with ice-cold acetone and centrifuged during 5 min to 13000g at 4°C. Precipitates were solved in PBS with 1% SDS, 1% triton X-100 and Complete Mini (EDTA free) during 30 min on ice. Protein concentration was quantified with Coomasie^®^ Plus Protein Assay Reagent following the manufacturer's protocol. 10 μg of protein were analyzed by NuPAGE™ 10% Bis-Tris Gels (Invitrogen) and separated proteins transferred to PVDF membranes (Millipore).

The antibodies used were mouse mAb against human Grp94, Hsp90, Hsc70/Hsp70, Hsp60, Hsp40 and TCP-1α (SPA-850, SPA-830, SPA-820, SPA-806, SPA-450 and CTA-122, Stressgen Biotechnologies, Victoria, Canada), Rab5 and Lck (BD Biosciences), β-actin, α-tubulin (clone AC-15 and clone B-5-1-2, Sigma), and α-actin (Santa Cruz, sc-1615). CD63 was detected using the mAb TEA3/18, a kind gift from Dr. Noa Martín-Cófreces, from Fundación CNIC, Madrid. Apo2L/TRAIL was revealed using the rabbit polyclonal antibody C35, kind gift from Avi Ashkenazi, Genentech, South San Francisco, USA. FasL was detected in cell and exosome extracts using a rabbit polyclonal antibody produced previously in our laboratory using cDNA immunization [71]. VCP was detected in cell and exosome extracts using a rabbit polyclonal antibody from Cell Signalling (Barcelona, Spain), especially optimized for immunoblot. Immunoblots were revealed by enhanced chemiluminiscence (Pierce, USA).

### Protein identification by mass spectrometry

#### MALDI-TOF

30 μg protein were runned in the same conditions as for silver staining. Gels were fixed with 50% methanol and 2% o-phosphoric acid over night. After that, gels were washed three times with distilled water during 20 min and incubated with 34% methanol, 2% o-phosphoric acid and 17% ammonium sulfate during 1 hour. Gels were stained with 34% methanol, 2% o-phosphoric acid, 17% ammonium sulfate and 0,066% Coomasie G-250 during at least 5 days. Finally, gels were distained with 25% methanol during 1 min and kept in distilled water until excising. Identified spots were excised from the gel and spotted on a MALDI AnchorChip target plate according to the manufacturer's instructions. Analysis was performed on an OrbitrapXL MALDI TOF instrument (Thermofisher) using Bruker Flex-control, Flex-analysis, and Biotools software. Calibration was performed externally using SIGMA Proteo- Mass™ Peptide MALDI–MS Calibration Kit. MALDI mass spectra were recorded within an m/z range of 700–3500 in the positive ion reflector mode. Mass spectra were obtained by averaging up to 300 individual laser shots. Each analysis was performed in triplicate.

#### LCMSMS

Protein samples were digested overnight at 37°C with 60 ng/μl trypsin in 50 mM ammonium bicarbonate, pH 8.8, containing 10% acetonitrile and 0.01% 5-cyclohexyl-1-pentyl-β-D maltoside. Tryptic peptide mixtures were separated using a nanoAcquity UPLC system (Waters GmbH, Eschborn, Germany). The nano UPLC system was coupled online to an LTQ Orbitrap XL mass spectrometer (Thermo Scientific, Bremen, Germany). Data were acquired by scan cycles of one FTMS scan with a resolution of 60000 at 400 m/z and a range from 370 to 2000 m/z in parallel with six MS/MS scans in the ion trap of the most abundant precursor ions. The mgf-files were used for database searches with the MASCOT search engine (Matrix Science, London, UK). Using this software, *P*< 0.05 was used as the detection criteria taken for the protein identification, meaning that all proteins identified has a probability of less than 5% to be false positives.

### Bioinformatics treatment of data

Using the uniprot ID numbers obtained, the protein name, species and function, together with their inclusion in different metabolic pathways was analyzed using the David software (http://david.abcc.ncifcrf.gov/list.jsp). Interaction profiles between the different proteins identified were represented using the STRING software (http://string-db.org/), and the significance for clustering in a given group was also calculated, being *P*≤ 0.012 for all the interaction nodes identified.

### Functional assays performed with the DBeQ VCP inhibitor

To estimate the functional value of VCP expression in the exosomes of tumoral Jurkat cells but not in those from normal T cell blasts, we performed several functional assays using the VCP inhibitor *N^2^, N^4^-*dibenzylquinazoline-2,4-diamine, also termed DBeQ [61].

#### Dose-response effect of DBeQ on cell growth and cell viability

Jurkat cells or human 6-day T-cell blasts were incubated overnight with increasing doses of DBeQ, from 3 to 6 μM, and cellular growth in comparison with non-treated control cells estimated by the MTT method, and cell death determined by Trypan blue staining and counting in an hemocytometer. Previously to establish this range of concentrations, which are not extremely toxic, DBeQ concentrations from 1 to 30 μM were tested.

#### Effect of DBeQ on cytotoxic exosome secretion from Jurkat or T cell blasts

To estimate the effect of DBeQ on this functional response, we used a bioassay previously optimized by our group [55, 56]. As controls, we performed also assays using the agonistic anti-Fas mAb CH11 (Upstate, Lake Placid, USA) at 50 ng/ml or recombinant TRAIL produced in our laboratory (e.g., see ref. 31) at 1 μg/ml, in the presence or absence of 3 μM DBeQ and/or the general caspase inhibitor Z-VAD-fmk (Bachem, Bubendorf, Switzerland) at 30 μM. For supernatant testing, Jurkat cells or T cell blasts were preincubated or not during 16h with 3 μM DBeQ, and, after washing out the inhibitor, cells were stimulated with 10 ng/ml of phorbol myristate-acetate (PMA) plus 600 nM ionomycin during 2h. Cell supernatants of stimulated cells were recovered by centrifugation, and tested overnight against non-activated Jurkat cells, as indicated in [56]. Cell death was estimated in these experiments by nuclear staining of dead cells with 7-amino-actinomycin D (7-AAD) and flow cytometry. In our previous studies we have shown that cytotoxicity on Jurkat cells of these supernatants is mainly due to FasL and Apo2L/TRAIL secretion associated with exosomes [8, 56, 57], being thus a functional test of exosome secretion.

## SUPPLEMENTARY FIGURE AND TABLE





## Abbreviations

MVB: multivesicular body
VCP: valosin-containing protein
DBeQ: *N^2^, N^4^-*dibenzylquinazoline-2,4-diamine
WAS: Wiskott-Aldrich syndrome
CTL: cytotoxic T lymphocytes
NK: natural killer
PHA: phytohemagglutinin
IEM: immunoelectron microscopy
MALDI-TOF: matrix-assisted laser desorption/Ionization with time-of-flight
MVP: major vault protein
MCM7: minichromosome maintenance protein 7
HNRPA2B1: heterogeneous nuclear riboprotein A2/B1
YBX-1: Y-box binding protein-1
LCMSMS: liquid chromatography – mass spectrometry x2
PMA: phorbol myristate acetate
TCR: T cell receptor
PBMC: peripheral blood mononuclear cells
TLC: thin layer chromatography

## References

[1] Schrier SL, Godin D, Gould RG, Swyryd B, Junga I, Seeger M (1971). Characterization of microvesicles produced by shearing of human erythrocyte membranes. Biochim Biophys Acta.

[2] Harding CV, Heuser JE, Stahl H (1983). Receptor-mediated endocytosis of transferrin and recycling of the transferrin receptor in rat reticulocytes. J Cell Biol.

[3] Pan BT, Johnstone RM (1983). Fate of the transferrin receptor during maturation of sheep reticulocytes in vitro: selective externalization of the receptor. Cell.

[4] Johnstone RM, Ahn J (1990). A common mechanism may be involved in the selective loss of plasma membrane functions during reticulocyte maturation. Biochim Biophys Acta.

[5] Dalton AJ (1975). Microvesicles and vesicles of multivesicular bodies verus “virus-like” particles. J Natl Cancer Inst.

[6] Der JE, Dixon WT, Jimbow K, Horikoshi T (1993). A murine monoclonal antibody against a melanosomal component highly expressed in early stages and common to normal and neoplastic melanocytes. Br J Cancer.

[7] Peters PJ, Borst J, Oorschot V, Fukuda M, Krähenbühl O, Tschopp J, Slot JW, Geuze HJ (1991). Cytotoxic T lymphocyte granules are secretory lysosomes, containing both perforin and granzymes. J Exp Med.

[8] Martínez-Lorenzo MJ, Anel A, Gamen S, Monleón I, Lasierra P, Larrad L, Piwñeiro A, Alava MA, Naval J (1999). Activated human T cells release bioactive Fas ligand and APO2 ligand in microvesicles. J Immunol.

[9] Raposo G, Nijman HW, Stoorvogel W, Leijendekker R, Harding CV, Melief CJM, Geuze HJ (1996). B lymphocytes secrete antigen-presenting vesicles. J Exp Med.

[10] Zitvogel L, Regnault A, Lozier A, Wolfers J, Flament C, Tenza D, Ricciardi-Castagnoli P, Raposo G, Amigorena S (1998). Eradication of established murine tumors using a novel cell-free vaccine : dendritic cell-derived exosomes. Nature Med.

[11] Denzer K, van Eijk M, Kleijmeer MJ, Jakobson E, de Groot C, Geuze HJ (2000). Follicular dendritic cells carry MHC class II-expressing microvesicles at their surface. J Immunol.

[12] Heijnen HF, Schiel AE, Fijnheer R, Geuze HJ, Sixma JJ (1999). Activated platelets release two types of membrane vesicles : microvesicles by surface shedding and exosomes derived from exocytosis of multivesicular bodies and alpha-granules. Blood.

[13] Skokos d, Le Panse S, Villa I, Rousselle JC, Peronet R, David B, Namame A, Mécheri S (2001). Mast cell-dependent B and T lymphocyte activation is mediated by the secretion of immunologically active exosomes. J Immunol.

[14] Abrahams VM, Straszewski-Chavez SL, Guller S, Mor G (2004). First trimester trophoblast cells secrete Fas ligand which induces immune cell apoptosis. Mol Human Reprod.

[15] van Niel G, Raposo G, Candalh C, Boussac M, Hershberg R, Cerf-Bensussan N, Heyman M (2001). Intestinal epithelial cells secrete exosome-like vesicles. Gastroenterol.

[16] Nilsson BO, Jin M, Einarsson B, Persson BE, Ronquist G (1998). Monoclonal antibodies against human prostasomes. Prostate.

[17] McKechnie NM, King BC, Fletcher E, Braun G (2006). Fas-ligand is stored in secretory lysosomes of ocular barrier epithelia and released with microvesicles. Exp Eye Res.

[18] Baranov V, Yeung MM, Hammarström S (1994). Expression of carcinoembrionic antigen and nonspecific cross-reacting 50 kDa antigen in human normal and cancerous colon mucosa: comparative ultrastructural study with monoclonal antibodies. Cancer Res.

[19] Oshima K, Aoki N, Kato T, Kitajima K, Matsuda T (2002). Secretion of a peripheral membrane protein, MFG-E8, as a complex with membrane vesicles. A possible role in membrane secretion. Eur J Biochem.

[20] Pisitkun T, Shen RF, Knepper MA (2004). Identification and proteomic profiling of exosomes in human urine. Proc Natl Acad Sci USA.

[21] Admyre C, Johansson SM, Qazi KR, Filén JJ, Lahesmaa R, Norman M, Neve EPA, Scheynius A, Gabrielson S (2007). Exosomes with Immune Modulatory Features Are Present in Human Breast Milk. J Immunol.

[22] Simpson RJ, Lim JWE, Moritz RL, Mathivanan S (2009). Exosomes: proteomic insights and diagnostic potential. Expert Rev Proteomics.

[23] Raposo G, Stoorvogel W (2013). Extracellular vesicles: Exosomes, microvesicles, and friends. J Cell Biol.

[24] Gutiérrez-Vázquez C, Villarroya-Beltri C, Mittelbrunn M, Sánchez-Madrid F (2013). Transfer of extracellular vesicles during immune cell-cell interactions. Immunol Rev.

[25] Monleón I, Martínez-Lorenzo MJ, Monteagudo L, Lasierra P, Taulés M, Iturralde M, Piñeiro A, Larrad L, Alava MA, Naval J, Anel A (2001). Differential secretion of Fas ligand- or APO2 ligand/TRAIL-carrying microvesicles during activation-induced death of human T cells. J Immunol.

[26] Alonso R, Mazzeo C, Rodriguez MC, Marsh M, Fraile-Ramos A, Calvo V, Avila-Flores A, Merida I, Izquierdo M (2011). Diacylglycerol kinase a regulates the formation and polarisation of mature multivesicular bodies involved in the secretion of Fas ligand-containing exosomes in T lymphocytes. Cell Death Differ.

[27] Robbins PD, Morelli AE (2014). Regulation of immune responses by extracellular vesicles. Nature Rev Immunol.

[28] Nikolov NP, Shimizu M, Cleland S, Balley D, Aoki J, Strom T, Schwartzberg PL, Candotti F, Siegel RM (2010). Systemic autoimmunity and defective Fas ligand secretion in the absence of the Wiskott-Aldrich syndrome protein. Blood.

[29] Martín-Fernández JM, Cabanillas JA, Rivero-Carmena M, Lacasa E, Pardo J, Anel A, Ramírez-Duque PR, Merino F, Rodríguez-Gallego C, Regueiro JR (2005). Herpesvirus saimiri-transformed CD8+ T cells as a tool to study Chediak-Higashi syndrome cytolytic lymphocytes. J Leukoc Biol.

[30] Martínez-Lorenzo MJ, Anel A, Sáez-Gutierrez B, Royo-Cañas M, Bosque A, Alava MA, Piñeiro A, Lasierra P, Asín-Ungría J, Larrad L (2007). Rheumatoid synovial fluid T cells are sensitive to APO2L/TRAIL. Clin Immunol.

[31] Martinez-Lostao L, García-Alvarez F, Basáñez G, Alegre-Aguarón E, Desportes P, Larrad L, Naval J, Martínez-Lorenzo MJ, Anel A (2010). Liposome-Bound APO2L/TRAIL Is an Effective Treatment in a Rabbit Model of Rheumatoid Arthritis. Arthrit Rheum.

[32] Lugini L, Ceccheti S, Huber V, Luciani F, Macchia G, Spadaro F, Paris L, Abalsamo L, Colone M, Molinari A (2013). Immune surveillance properties of human NK cell-derived exosomes. J Immunol.

[33] Frangsmyr L, Baranov V, Nagaeva O, Stendahl U, Kjellberg L, Mincheva-Nilsson L (2005). Cytoplasmic microvesicular form of Fas ligand in human early placenta: switching the tissue immune privilege hypothesis from cellular to vesicular level. Mol Human Reprod.

[34] André F, Schartz NEC, Movassagh M, Flament C, Pautier P, Morice P, Pomel C, Lhomme C, Escudier B, Le Chevalier T, Tusz T, Amigorena S, Raposo G, Angevin E, Zitvogel L (2002). Malignant effusions and immunogenic tumour-derived exosomes. Lancet.

[35] Kim SH, Bianco N, Menon R, Lechman ER, Shufesky WJ, Morelli AE, Robbins PD (2005). Exosomes Derived from Genetically Modified DC Expressing FasL Are Anti-inflammatory and Immunosuppressive. Mol Ther.

[36] Huber V, Fais S, Iero M, Lugini L, Canese P, Squarcina P, Zaccheddu A, Colone M, Arancia G, Gentile M (2005). Human colorectal cancer cells induce T-cell death through release of proapoptotic microvesicles: role in immune escape. Gastroenterol.

[37] Abrahams VM, Straszewski SL, Kamsteeg M, Hanczaruk B, Schwartz PE, Rutherford TJ, Mor G (2003). Epithelial ovarian cancer cells secrete functional Fas ligand. Cancer Res.

[38] Taylor DD, Gerçel-Taylor C, Lyons KS, Stanson J, Whiteside TL (2003). T-Cell Apoptosis and Suppression of T-Cell Receptor/CD3-z by Fas Ligand-Containing Membrane Vesicles Shed from Ovarian Tumors. Clin Cancer Res.

[39] Andreola G, Rivoltini L, Castelli C, Huber V, Perego P, Deho P, Squarcina P, Accornero P, Lozupone F, Lugini L (2002). Induction of lymphocyte apoptosis by tumor cell secretion of FasL-bearing microvesicles. J Exp Med.

[40] Martínez-Lorenzo MJ, Anel A, Alava MA, Piñeiro A, Naval J, Lasierra P, Larrad L (2004). The human melanoma cell line MelJuso secretes bioactive FasL and APO2L/TRAIL on the surface of microvesicles. Possible contribution to tumor counterattack. Exp Cell Res.

[41] Valenti R, Huber V, Iero M, Filipazzi P, Parmiani G, Rivoltini L (2007). Tumor-released microvesicles as vehicles of immunosupression. Cancer Res.

[42] Wieckowski EU, Visus C, Szajnik M, Szczepanski MJ, Storkus WJ, Whiteside TL (2009). Tumor-Derived Microvesicles Promote Regulatory T Cell Expansion and Induce Apoptosis in Tumor-Reactive Activated CD8^+^ T Lymphocytes. J Immunol.

[43] Wubbolts RW, Leckie RS, Veenhuizen PTM, Schwarzmann G, Moebius W, Hoernchemeyer J, Slot JW, Geuze HJ, Stoorvogel W (2003). Proteomic and biochemical analyses of human B cell-derived exosomes : Potential implications for their function and multivesicular body formation. J Biol Chem.

[44] Théry C, Boussac M, Véron P, Ricciardi-Castagnoli P, Raposo G, Garin J, Amigorena S (2001). Proteomic analysis of dendritic cell-derived exosomes : A secreted subcellular compartment distinct from apoptotic vesicles. J Immunol.

[45] Bard MP, Hegmans JP, Hemmes A, Luider TM, Willemsen R, Severijnen LA, van Meerbeeck JP, Burgers SA, Hoogsteden HC, Lambrecht BN (2004). Proteomic analysis of exosomes isolated from malignant pleural effusions. Am J Respir Cell Mol Biol.

[46] Mathivanan S, Simpson RJ (2009). ExoCarta: a compendium of exosomal proteins and RNA. Proteomics.

[47] Kalra H, Simpson RJ, Ji H, Aikawa E, Altevogt P, Askenase P, Bond VC, Borrás FE, Breakefield X, Budnik V (2012). Vesiclepedia: a compendium for extracellular vesicles with continuous community annotation. PLos Biol.

[48] Valadi H, Ekström K, Bossios A, Sjöstrand M, Lee JJ, Lötvall JO (2007). Exosome-mediated transfer of mRNAs and microRNAs is a novel mechanism of genetic exchange between cells. Nature Cell Biol.

[49] Mittelbrunn M, Gutiérrez-Vázquez C, Villarroya-Beltri C, González S, Sánchez-Cabo F, González MA, Bernad A, Sánchez-Madrid F (2011). Unidirectional transfer of microRNnA-loaded exosomes from T cells to antigen-presenting cells. Nature Comm.

[50] Mittelbrunn M, Sánchez-Madrid F (2012). Intercellular communication: diverse structures for exchange of genetic information. Nature Rev Mol Cell Biol.

[51] Blanchard N, Lankar D, Faure F, Regnault A, Dumont C, Raposo G, Hivroz C (2002). TCR activation of human T cells induces the production of exosomes bearing the TCR/CD3/z complex. J Immunol.

[52] Villarroya-Beltri C, Gutiérrez-Vázquez C, Sánchez-Cabo F, Pérez-Hernández D, Vázquez J, Martín-Cofreces N, Martínez-Herrera D, Pascual-Montano A, Mittelbrunn M, Sánchez-Madrid F (2013). Sumoylated hnRNPA2B1 controls the sorting of miRNAs into exosomes through binding to specific motifs. Nature Commun.

[53] Wang Q, Song C, Li CCH (2004). Molecular perspectives on p97–VCP: progress in understanding its structure and diverse biological functions. J Struct Biol.

[54] Yamamoto S, Tomita Y, Hoshida Y, Takiguchi S, Fujiwara Y, Yasuda T, Yano M, Nakamori S, Sakon M, Monden M, Aozasa K (2003). Expression Level of Valosin-Containing Protein Is Strongly Associated With Progression and Prognosis of Gastric Carcinoma. J Clin Oncol.

[55] Del Rey M, Ruiz-Contreras J, Bosque A, Calleja S, Gómez-Rial J, Roldán E, Morales P, Anel A, Paz-Artal E, Allende LM (2006). A homozygous Fas ligand gene mutation in a patient causes a new type of autoimmune lymphoproliferative syndrome. Blood.

[56] Martínez-Lorenzo MJ, Alava MA, Anel A, Piñeiro A, Naval J (1996). Release of preformed Fas ligand in soluble form is the major factor for activation-induced death of Jurkat T cells. Immunology.

[57] Martínez-Lorenzo MJ, Alava MA, Gamen S, Kim JK, Chuntharapai A, Piñeiro A, Naval J, Anel A (1998). Involvement of APO2 ligand/TRAIL in activation-induced death of Jurkat and human peripheral blood T cells. Eur J Immunol.

[58] Stenqvist A, Nagaeva O, Baranov V, Mincheva-Nilsson L (2013). Exosomes Secreted by Human Placenta Carry Functional Fas Ligand and TRAIL Molecules and Convey Apoptosis in Activated Immune Cells, Suggesting Exosome-Mediated Immune Privilege of the Fetus. J Immunol.

[59] Schmidt H, Gelhaus C, Nebendahl M, Lettau M, Lucius R, Leippe M, Kabelitz D, Janssen O (2011). Effector Granules in Human T Lymphocytes: Proteomic Evidence for Two Distinct Species of Cytotoxic Effector Vesicles. J Proteome Res.

[60] Pérez-Hernández D, Gutiérrez-Vázquez C, Jorge I, López-Martín S, Ursa A, Sánchez-Madrid F, Vázquez J, Yáñez-Mó M (2013). The Intracellular Interactome of Tetraspanin-enriched Microdomains Reveals Their Function as Sorting Machineries toward Exosomes. J Biol Chem.

[61] Chou TF, Brown SJ, Minond D, Nordin BE, Li K, Jones AC, Chase P, Porubsky PR, Stoltz BM, Schoenen FJ (2011). al. e. Reversible inhibitor of p97, DBeQ, impairs both ubiquitin- dependent and autophagic protein clearance pathways. Proc Natl Acad Sci USA.

[62] Yi P, Higa A, Taouji S, Bexiga MG, Marza E, Arma D, Castain C, Le Bail B, Simpson JC, Rosenbaum J (2012). Sorafenib-Mediated Targeting of the AAA^+^ ATPase p97/VCP Leads to Disruption of the Secretory Pathway, Endoplasmic Reticulum Stress, and Hepatocellular Cancer Cell Death. Mol Cancer Ther.

[63] Melo S, Luecke L, Kahlert C, Fernandez A, Gammon S, Kaye J, LeBleu V, Mittendorf E, Weitz J, Rahbari N (2015). Glypican-1 identifies cancer exosomes and detects early pancreatic cancer. Nature.

[64] Peinado H, Alečković M, Lavotshkin S, Matei I, Costa-Silva B, Moreno-Bueno G, Hergueta-Redondo M, Williams C, García-Santos G, Nitadori-Hoshino A (2012). Melanoma exosomes educate bone marrow progenitor cells toward a pro-metastatic phenotype through MET. Nature Med.

[65] Mantwill K, Köhler-Vargas N, Bernshausen A, Bieler A, Lage H, Kaszubiak A, Surowiak P, Dravits T, Treiber U, Hartung R (2006). Inhibition of the Multidrug-Resistant Phenotype by Targeting YB-1 with a Conditionally Oncolytic Adenovirus: Implications for Combinatorial Treatment Regimen with Chemotherapeutic Agents. Cancer Res.

[66] Luo J (2011). Oncogenic activity of MCM7 transforming cluster. World J Clin Oncol.

[67] Bosque A, Pardo J, Martínez-Lorenzo MJ, Iturralde M, Marzo I, Piñeiro A, Alava MA, Naval J, Anel A (2005). Down-regulation of normal human T cell blast activation: roles of APO2L/TRAIL, FasL and c- FLIP, Bim or Bcl-x isoform expression. J Leukoc Biol.

[68] Kobayashi T, Stang E, Fang KS, de Moerlosse P, Parton RG, Gruenberg J (1998). A lipid associated with the antiphospholipid syndrome regulates endosome structure and function. Nature.

[69] Iturralde M, Pardo J, Lacasa E, Barrio G, Alava MA, Piñeiro A, Naval J, Anel A (2005). Characterization of the lipolytic pathways that mediate free fatty acid release during Fas/CD95-induced apoptosis. Apoptosis.

[70] Blum H, Beier H, Gross HJ (1987). Improved silver staining of plant proteins, RNA and DNA in polyacrylamide gels. Electrophoresis.

[71] Diestre C, Martínez-Lorenzo MJ, Bosque A, Naval J, Larrad L, Anel A (2006). Generation of rabbit antibodies against death ligands by cDNA immunization. J Immunol Meth.

