# Supplementary material for: Comparative proteomics of exosomes secreted by tumoral Jurkat T cells and normal human T cell blasts unravels a potential tumorigenic role for valosin-containing protein
Source: Oncotarget. 2016 Apr 11;7(20):29287–305. doi: 10.18632/oncotarget.8678 (PMC5045396; doi:10.18632/oncotarget.8678)
Supplement: Supplementary file 1 [file oncotarget-07-29287-s001.pdf]

SUPPLEMENTARY FIGURE AND TABLE

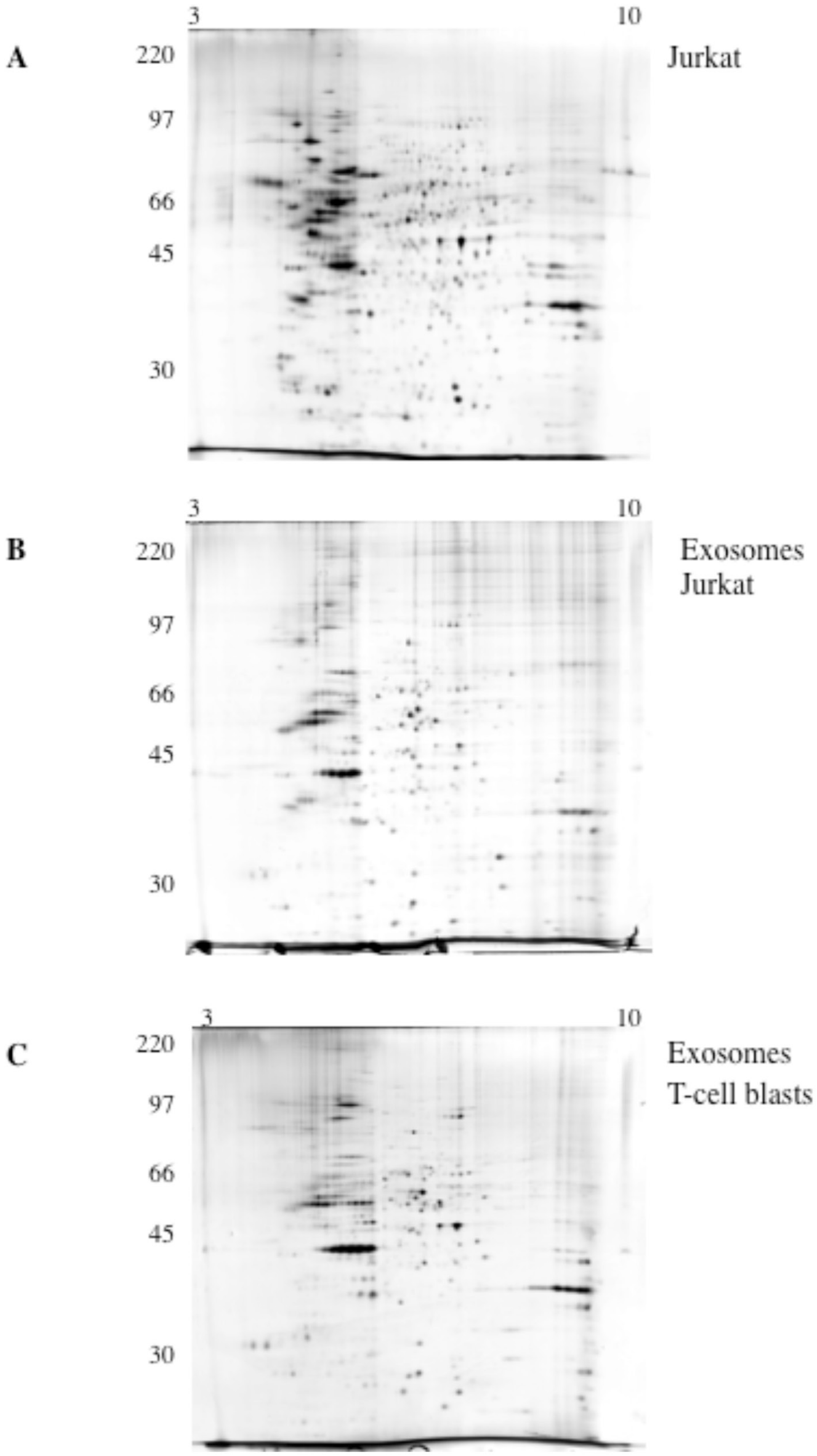

Supplementary Figure S1: 2D separation of proteins from cells or from exosomes. 2D gels were performed on samples of 30 µg total protein extracted from Jurkat cells (upper panel), from Jurkat exosomes (middle panel) or from T cell blast exosomes (lower panel) and protein spots were silver-stained. Isoelectric point is indicated above and molecular weight on the left. The images are representative of at least 5 different gels performed for each type of sample.

Supplementary Table S1: Proteins detected in exosomes

See Supplementary File 1
