# Supplementary material for: Comparative proteomics of exosomes secreted by tumoral Jurkat T cells and normal human T cell blasts unravels a potential tumorigenic role for valosin-containing protein
Source: Oncotarget. 2016 Apr 11;7(20):29287–305. doi: 10.18632/oncotarget.8678 (PMC5045396; doi:10.18632/oncotarget.8678)
Supplement: Supplementary file 2 [file oncotarget-07-29287-s002.doc]

**Suppl. Table 1. Proteins detected in exosomes**

**Shared proteins**

UNIPROT_ACCESSION Name Species

P25398 ribosomal protein S12; ribosomal protein S12 pseudogene 4; ribosomal protein

S12 pseudogene 11; ribosomal protein S12 pseudogene 9 Homo sapiens

Q9NQC3 reticulon 4 Homo sapiens

O75494 FUS interacting protein (serine/arginine-rich) 1; similar to FUS interacting

protein (serine-arginine rich) 1 Homo sapiens

P11940 poly(A) binding protein, cytoplasmic pseudogene 5; poly(A) binding protein,

cytoplasmic 1 Homo sapiens

Q92522 H1 histone family, member X Homo sapiens

P49368 chaperonin containing TCP1, subunit 3 (gamma) Homo sapiens

P23246 splicing factor proline/glutamine-rich (polypyrimidine tract binding protein

associated) Homo sapiens

P06702 S100 calcium binding protein A9 Homo sapiens

P31942 heterogeneous nuclear ribonucleoprotein H3 (2H9) Homo sapiens

P63261 actin, gamma 1 Homo sapiens

O60506 synaptotagmin binding, cytoplasmic RNA interacting proteinHomo sapiens

P62841 ribosomal protein S15 pseudogene 5; ribosomal protein S15 Homo sapiens

O75340 aryl-hydrocarbon receptor repressor; programmed cell death 6Homo sapiens

Q9Y2W2 WW domain binding protein 11 Homo sapiens

Q15125 emopamil binding protein (sterol isomerase) Homo sapiens

P05556 integrin, beta 1 (fibronectin receptor, beta polypeptide, antigen CD29

includes MDF2, MSK12) Homo sapiens

Q15007 Wilms tumor 1 associated protein Homo sapiens

P61158 ARP3 actin-related protein 3 homolog (yeast) Homo sapiens

P50995 annexin A11 Homo sapiens

Q15758 solute carrier family 1 (neutral amino acid transporter), member 5

Homo sapiens

Q92804 TAF15 RNA polymerase II, TATA box binding protein (TBP)-associated factor,

68kDa Homo sapiens

P62081 ribosomal protein S7; ribosomal protein S7 pseudogene 11; ribosomal protein

S7 pseudogene 4; ribosomal protein S7 pseudogene 10 Homo sapiens

Q92520 family with sequence similarity 3, member C Homo sapiens

P05386 ribosomal protein, large, P1 Homo sapiens

Q9BVC6 transmembrane protein 109 Homo sapiens

P09429 high-mobility group box 1; high-mobility group box 1-like 10Homo sapiens

P08133 annexin A6 Homo sapiens

P62277 ribosomal protein S13 pseudogene 8; ribosomal protein S13; ribosomal protein

S13 pseudogene 2 Homo sapiens

P68363 hypothetical gene supported by AF081484; NM_006082; tubulin, alpha 1b

Homo sapiens

Q14103 heterogeneous nuclear ribonucleoprotein D (AU-rich element RNA binding

protein 1, 37kDa) Homo sapiens

P81605 dermcidin Homo sapiens

P38919 eukaryotic translation initiation factor 4A, isoform 3 Homo sapiens

Q15459 splicing factor 3a, subunit 1, 120kDa Homo sapiens

Q08722 CD47 molecule Homo sapiens

Q9H307 pinin, desmosome associated protein Homo sapiens

P09661 small nuclear ribonucleoprotein polypeptide A' Homo sapiens

Q99729 heterogeneous nuclear ribonucleoprotein A/B Homo sapiens

Q9ULR0 ISY1 splicing factor homolog (S. cerevisiae) Homo sapiens

P22626 heterogeneous nuclear ribonucleoprotein A2/B1 Homo sapiens

P08579 small nuclear ribonucleoprotein polypeptide B'' Homo sapiens

P62308 hypothetical protein LOC100132425; similar to small nuclear

ribonucleoprotein polypeptide G; small nuclear ribonucleoprotein polypeptide

G; small nuclear ribonucleoprotein G-like protein Homo sapiens

P07237 prolyl 4-hydroxylase, beta polypeptide Homo sapiens

Q969G3 SWI/SNF related, matrix associated, actin dependent regulator of chromatin,

subfamily e, member 1 Homo sapiens

P02768 albumin Homo sapiens

Q15428 splicing factor 3a, subunit 2, 66kDa Homo sapiens

P78371 chaperonin containing TCP1, subunit 2 (beta) Homo sapiens

P27701 CD82 molecule Homo sapiens

P60660 myosin, light chain 6, alkali, smooth muscle and non-muscleHomo sapiens

Q12905 interleukin enhancer binding factor 2, 45kDa Homo sapiens

P18124 ribosomal protein L7 pseudogene 26; ribosomal protein L7 pseudogene 16;

ribosomal protein L7; ribosomal protein L7 pseudogene 32; ribosomal protein

L7 pseudogene 23; ribosomal protein L7 pseudogene 24; ribosomal protein L7

pseudogene 20 Homo sapiens

P52597 heterogeneous nuclear ribonucleoprotein F Homo sapiens

P38159 similar to RNA binding motif protein, X-linked; similar to hCG2011544; RNA

binding motif protein, X-linked Homo sapiens

P06748 nucleophosmin 1 (nucleolar phosphoprotein B23, numatrin) pseudogene 21;

hypothetical LOC100131044; similar to nucleophosmin 1; nucleophosmin

(nucleolar phosphoprotein B23, numatrin) Homo sapiens

P60228 eukaryotic translation initiation factor 3, subunit E Homo sapiens

P09651 heterogeneous nuclear ribonucleoprotein A1-like 3; similar to heterogeneous

nuclear ribonucleoprotein A1; heterogeneous nuclear ribonucleoprotein A1

pseudogene 2; heterogeneous nuclear ribonucleoprotein A1; heterogeneous

nuclear ribonucleoprotein A1 pseudogene Homo sapiens

Q14761 protein tyrosine phosphatase, receptor type, C-associated protein

Homo sapiens

P63162 small nuclear ribonucleoprotein polypeptide N; SNRPN upstream reading frame

Homo sapiens

O14828 secretory carrier membrane protein 3 Homo sapiens

P04083 annexin A1 Homo sapiens

Q5D862 filaggrin family member 2 Homo sapiens

Q06323 proteasome (prosome, macropain) activator subunit 1 (PA28 alpha)

Homo sapiens

P60900 proteasome (prosome, macropain) subunit, alpha type, 6 Homo sapiens

Q10589 NPC-A-7; bone marrow stromal cell antigen 2 Homo sapiens

Q14764 major vault protein Homo sapiens

P31943 heterogeneous nuclear ribonucleoprotein H1 (H) Homo sapiens

P13473 lysosomal-associated membrane protein 2 Homo sapiens

P35579 myosin, heavy chain 9, non-muscle Homo sapiens

P07355 annexin A2 pseudogene 3; annexin A2; annexin A2 pseudogene 1Homo sapiens

P16150 sialophorin Homo sapiens

P62906 ribosomal protein L10a pseudogene 6; ribosomal protein L10a; ribosomal

protein L10a pseudogene 9 Homo sapiens

P60033 CD81 molecule Homo sapiens

P25787 proteasome (prosome, macropain) subunit, alpha type, 2 Homo sapiens

P61978 heterogeneous nuclear ribonucleoprotein K; similar to heterogeneous nuclear

ribonucleoprotein K Homo sapiens

P49755 transmembrane emp24-like trafficking protein 10 (yeast) Homo sapiens

Q13247 splicing factor, arginine/serine-rich 6; similar to arginine/serine-rich

splicing factor 6 Homo sapiens

P62750 ribosomal protein L23a pseudogene 63; ribosomal protein L23a pseudogene 75;

ribosomal protein L23a pseudogene 37; ribosomal protein L23a pseudogene 65;

ribosomal protein L23a pseudogene 43; ribosomal protein L23a pseudogene 44;

ribosomal protein L23a Homo sapiens

Q12906 interleukin enhancer binding factor 3, 90kDa Homo sapiens

Q00839 heterogeneous nuclear ribonucleoprotein U (scaffold attachment factor A)

Homo sapiens

P19397 CD53 molecule Homo sapiens

P05387 ribosomal protein, large, P2 pseudogene 3; ribosomal protein, large, P2

Homo sapiens

P20618 proteasome (prosome, macropain) subunit, beta type, 1 Homo sapiens

P08865 ribosomal protein SA pseudogene 9; ribosomal protein SA pseudogene 8;

ribosomal protein SA pseudogene 58; ribosomal protein SA pseudogene 19;

ribosomal protein SA pseudogene 18; ribosomal protein SA; ribosomal protein

SA pseudogene 15; ribosomal protein SA pseudogene 61; ribosomal protein SA

pseudogene 29; ribosomal protein SA pseudogene 12 Homo sapiens

Q15233 non-POU domain containing, octamer-binding Homo sapiens

Q13344 fusion (involved in t(12;16) in malignant liposarcoma) Homo sapiens

O00161 synaptosomal-associated protein, 23kDa Homo sapiens

P07437 tubulin, beta; similar to tubulin, beta 5; tubulin, beta pseudogene 2;

tubulin, beta pseudogene 1 Homo sapiens

P05388 ribosomal protein, large, P0 pseudogene 2; ribosomal protein, large, P0

pseudogene 3; ribosomal protein, large, P0 pseudogene 6; ribosomal protein,

large, P0 Homo sapiens

Q9Y230 RuvB-like 2 (E. coli) Homo sapiens

Q549M8 chromosome 14 open reading frame 166 Homo sapiens

P62888 ribosomal protein L30 Homo sapiens

P62241 ribosomal protein S8; ribosomal protein S8 pseudogene 8; ribosomal protein

S8 pseudogene 10 Homo sapiens

P13073 cytochrome c oxidase subunit IV isoform 1 Homo sapiens

P54709 ATPase, Na+/K+ transporting, beta 3 polypeptide Homo sapiens

O15145 similar to actin related protein 2/3 complex subunit 3; hypothetical

LOC729841; actin related protein 2/3 complex, subunit 3, 21kDaHomo sapiens

P52272 heterogeneous nuclear ribonucleoprotein M Homo sapiens

P20073 annexin A7 Homo sapiens

O15260 surfeit 4 Homo sapiens

P51572 B-cell receptor-associated protein 31 Homo sapiens

P30101 protein disulfide isomerase family A, member 3 Homo sapiens

P05141 solute carrier family 25 (mitochondrial carrier; adenine nucleotide

translocator), member 5; solute carrier family 25 (mitochondrial carrier;

adenine nucleotide translocator), member 5 pseudogene 8 Homo sapiens

O43493 trans-golgi network protein 2 Homo sapiens

P55145 mesencephalic astrocyte-derived neurotrophic factor Homo sapiens

P06744 glucose phosphate isomerase Homo sapiens

P06733 enolase 1, (alpha) Homo sapiens

P20645 mannose-6-phosphate receptor (cation dependent) Homo sapiens

P09525 annexin A4 Homo sapiens

O14818 proteasome (prosome, macropain) subunit, alpha type, 7 Homo sapiens

P27824 calnexin Homo sapiens

Q9UL46 proteasome (prosome, macropain) activator subunit 2 (PA28 beta)Homo sapiens

Q8TAQ2 SWI/SNF related, matrix associated, actin dependent regulator of chromatin,

subfamily c, member 2 Homo sapiens

P27797 calreticulin Homo sapiens

Q9UKM9 RNA binding protein, autoantigenic (hnRNP-associated with lethal yellow

homolog (mouse)) Homo sapiens

P14314 protein kinase C substrate 80K-H Homo sapiens

P80303 nucleobindin 2 Homo sapiens

P08670 vimentin Homo sapiens

O75533 splicing factor 3b, subunit 1, 155kDa Homo sapiens

P49721 proteasome (prosome, macropain) subunit, beta type, 2 Homo sapiens

P61247 ribosomal protein S3A pseudogene 5; ribosomal protein S3a pseudogene 47;

ribosomal protein S3a pseudogene 49; ribosomal protein S3A; hypothetical

LOC100131699; hypothetical LOC100130107 Homo sapiens

Q04941 proteolipid protein 2 (colonic epithelium-enriched) Homo sapiens

P60709 actin, beta Homo sapiens

P68104 eukaryotic translation elongation factor 1 alpha-like 7; eukaryotic

translation elongation factor 1 alpha-like 3; similar to eukaryotic

translation elongation factor 1 alpha 1; eukaryotic translation elongation

factor 1 alpha 1 Homo sapiens

P62851 ribosomal protein S25 pseudogene 8; ribosomal protein S25 Homo sapiens

P07910 heterogeneous nuclear ribonucleoprotein C (C1/C2) Homo sapiens

P39019 ribosomal protein S19 pseudogene 3; ribosomal protein S19 Homo sapiens

Q13573 SNW domain containing 1 Homo sapiens

Q15427 splicing factor 3b, subunit 4, 49kDa Homo sapiens

P46783 ribosomal protein S10; ribosomal protein S10 pseudogene 4; ribosomal protein

S10 pseudogene 11; ribosomal protein S10 pseudogene 22; ribosomal protein S10

pseudogene 7; ribosomal protein S10 pseudogene 13 Homo sapiens

P46782 ribosomal protein S5 Homo sapiens

P19338 nucleolin Homo sapiens

P09012 small nuclear ribonucleoprotein polypeptide A Homo sapiens

P61353 ribosomal protein L27 Homo sapiens

P62899 ribosomal protein L31 pseudogene 49; ribosomal protein L31 pseudogene 17;

ribosomal protein L31 Homo sapiens

Q15008 proteasome (prosome, macropain) 26S subunit, non-ATPase, 6Homo sapiens

P04406 glyceraldehyde-3-phosphate dehydrogenase-like 6; hypothetical protein

LOC100133042; glyceraldehyde-3-phosphate dehydrogenase Homo sapiens

P06753 tropomyosin 3 Homo sapiens

P30050 ribosomal protein L12 pseudogene 2; ribosomal protein L12 pseudogene 32;

ribosomal protein L12 pseudogene 35; ribosomal protein L12 pseudogene 19;

ribosomal protein L12 pseudogene 6; ribosomal protein L12; ribosomal protein

L12 pseudogene 14 Homo sapiens

P08758 annexin A5 Homo sapiens

P49720 proteasome (prosome, macropain) subunit, beta type, 3 Homo sapiens

P37802 transgelin 2 Homo sapiens

P08174 CD55 molecule, decay accelerating factor for complement (Cromer blood group)

Homo sapiens

Q14696 mesoderm development candidate 2 Homo sapiens

Q07955 splicing factor, arginine/serine-rich 1 Homo sapiens

P43243 matrin 3 Homo sapiens

**Proteins detected only in exosomes from T cell blasts**

UNIPROT_ACCESSION Name Species

Q9BVK6 transmembrane emp24 protein transport domain containing 9 Homo sapiens

Q8TF72 shroom family member 3 Homo sapiens

O15400 syntaxin 7 Homo sapiens

Q01105 SET nuclear oncogene; similar to SET translocation Homo sapiens

Q15041 ADP-ribosylation factor-like 6 interacting protein 1 Homo sapiens

Q53HL1 myosin, light chain 12A, regulatory, non-sarcomeric Homo sapiens

P19105 myosin, light chain 12A, regulatory, non-sarcomeric Homo sapiens

P62913 ribosomal protein L11 Homo sapiens

Q96AE4 far upstream element (FUSE) binding protein 1 Homo sapiens

P17948 fms-related tyrosine kinase 1 (vascular endothelial growth factor/vascular

permeability factor receptor) Homo sapiens

P34910 ecotropic viral integration site 2B Homo sapiens

O43390 heterogeneous nuclear ribonucleoprotein R Homo sapiens

Q8WYR6 family with sequence similarity 48, member A Homo sapiens

Q9H009 nascent polypeptide-associated complex alpha subunit 2 Homo sapiens

P62988 ubiquitin A-52 residue ribosomal protein fusion product 1 Homo sapiens

P09326 CD48 molecule Homo sapiens

P08195 solute carrier family 3 (activators of dibasic and neutral amino acid

transport), member 2 Homo sapiens

Q9NR30 DEAD (Asp-Glu-Ala-Asp) box polypeptide 21 Homo sapiens

Q92896 golgi apparatus protein 1 Homo sapiens

Q9Y2Q3 glutathione S-transferase kappa 1 Homo sapiens

O15143 actin related protein 2/3 complex, subunit 1B, 41kDa; similar to Actin-

related protein 2/3 complex subunit 1B (ARP2/3 complex 41 kDa subunit) (p41-

ARC) Homo sapiens

P51571 signal sequence receptor, delta (translocon-associated protein delta)

Homo sapiens

Q9UQ80 proliferation-associated 2G4, 38kDa; proliferation-associated 2G4 pseudogene

4 Homo sapiens

P20701 integrin, alpha L (antigen CD11A (p180), lymphocyte function-associated

antigen 1; alpha polypeptide) Homo sapiens

Q13435 splicing factor 3b, subunit 2, 145kDa Homo sapiens

Q01518 CAP, adenylate cyclase-associated protein 1 (yeast) Homo sapiens

O15173 progesterone receptor membrane component 2 Homo sapiens

Q99733 nucleosome assembly protein 1-like 4 Homo sapiens

P63244 guanine nucleotide binding protein (G protein), beta polypeptide 2-like 1

Homo sapiens

P19256 CD58 molecule Homo sapiens

Q02818 nucleobindin 1 Homo sapiens

P08779 keratin 16; keratin type 16-like Homo sapiens

O95400 CD2 (cytoplasmic tail) binding protein 2 Homo sapiens

P62937 similar to TRIMCyp; peptidylprolyl isomerase A (cyclophilin A);

peptidylprolyl isomerase A (cyclophilin A)-like 3 Homo sapiens

P33241 lymphocyte-specific protein 1 Homo sapiens

Q6PCB8 embigin homolog (mouse) Homo sapiens

P01891 major histocompatibility complex, class I, A Homo sapiens

P52907 capping protein (actin filament) muscle Z-line, alpha 1 Homo sapiens

Q9H7M9 chromosome 10 open reading frame 54 Homo sapiens

P46976 glycogenin 1 Homo sapiens

Q12846 syntaxin 4 Homo sapiens

Q09666 AHNAK nucleoprotein Homo sapiens

P50502 similar to heat shock 70kD protein binding protein; suppression of

tumorigenicity 13 (colon carcinoma) (Hsp70 interacting protein)Homo sapiens

P62847 ribosomal protein S24 Homo sapiens

P32942 intercellular adhesion molecule 3 Homo sapiens

Q01628 interferon induced transmembrane protein 3 (1-8U) Homo sapiens

Q14444 cell cycle associated protein 1 Homo sapiens

Q08188 transglutaminase 3 (E polypeptide, protein-glutamine-gamma-

glutamyltransferase) Homo sapiens

P26599 polypyrimidine tract binding protein 1 Homo sapiens

P62988 ribosomal protein S27a pseudogene 12; ribosomal protein S27a; ribosomal

protein S27a pseudogene 11; ribosomal protein S27a pseudogene 16Homo sapiens

Q16181 septin 7 Homo sapiens

P62424 ribosomal protein L7a pseudogene 70; ribosomal protein L7a; ribosomal

protein L7a pseudogene 30; ribosomal protein L7a pseudogene 66; ribosomal

protein L7a pseudogene 27; ribosomal protein L7a pseudogene 11; ribosomal

protein L7a pseudogene 62 Homo sapiens

Q96DU3 SLAM family member 6 Homo sapiens

P05107 integrin, beta 2 (complement component 3 receptor 3 and 4 subunit)

Homo sapiens

Q13451 FK506 binding protein 5 Homo sapiens

P29966 myristoylated alanine-rich protein kinase C substrate Homo sapiens

Q99613 eukaryotic translation initiation factor 3, subunit C Homo sapiens

P12956 X-ray repair complementing defective repair in Chinese hamster cells 6;

similar to ATP-dependent DNA helicase II, 70 kDa subunit Homo sapiens

Q9NW64 RNA binding motif protein 22 Homo sapiens

Q5JR94 ribosomal protein S8; ribosomal protein S8 pseudogene 8; ribosomal protein

S8 pseudogene 10 Homo sapiens

P08621 small nuclear ribonucleoprotein 70kDa (U1) Homo sapiens

O75390 citrate synthase Homo sapiens

P22392 non-metastatic cells 1, protein (NM23A) expressed in; NME1-NME2 readthrough

transcript; non-metastatic cells 2, protein (NM23B) expressed inHomo sapiens

P82650 mitochondrial ribosomal protein S22 Homo sapiens

P04899 guanine nucleotide binding protein (G protein), alpha inhibiting activity

polypeptide 2 Homo sapiens

Q9Y6X5 ectonucleotide pyrophosphatase/phosphodiesterase 4 (putative function)

Homo sapiens

P26583 high-mobility group box 2 Homo sapiens

Q15637 splicing factor 1 Homo sapiens

Q5W009 RNA binding motif protein 17 Homo sapiens

P50395 GDP dissociation inhibitor 2 Homo sapiens

P31946 tyrosine 3-monooxygenase/tryptophan 5-monooxygenase activation protein, beta

polypeptide Homo sapiens

P04075 aldolase A, fructose-bisphosphate Homo sapiens

P01857 immunoglobulin heavy constant gamma 1 (G1m marker); immunoglobulin heavy

constant mu; immunoglobulin heavy variable 3-7; immunoglobulin heavy

constant gamma 3 (G3m marker); immunoglobulin heavy variable 3-11

(gene/pseudogene); immunoglobulin heavy variable 4-31; immunoglobulin heavy

locus Homo sapiens

P15529 CD46 molecule, complement regulatory protein Homo sapiens

Q6NZ59 ATP synthase, H+ transporting, mitochondrial F0 complex, subunit F6

Homo sapiens

P14866 similar to heterogeneous nuclear ribonucleoprotein L-like; heterogeneous

nuclear ribonucleoprotein L Homo sapiens

Q6IB99 Ras association (RalGDS/AF-6) domain family (N-terminal) member 8

Homo sapiens

Q71UI9 H2A histone family, member V Homo sapiens

O00116 alkylglycerone phosphate synthase Homo sapiens

P26447 S100 calcium binding protein A4 Homo sapiens

Q5T749 keratinocyte proline-rich protein Homo sapiens

A2RU81 DEAD (Asp-Glu-Ala-Asp) box polypeptide 58 Homo sapiens

P62988 ubiquitin B Homo sapiens

Q59HE3 calpastatin Homo sapiens

P08575 protein tyrosine phosphatase, receptor type, C Homo sapiens

P11279 lysosomal-associated membrane protein 1 Homo sapiens

Q15393 splicing factor 3b, subunit 3, 130kDa Homo sapiens

P33240 cleavage stimulation factor, 3' pre-RNA, subunit 2, 64kDa Homo sapiens

P40227 chaperonin containing TCP1, subunit 6A (zeta 1) Homo sapiens

P48960 CD97 molecule Homo sapiens

Q14730 Sjogren syndrome antigen B (autoantigen La) Homo sapiens

P62988 ubiquitin C Homo sapiens

P0C0S8 histone cluster 1, H2ag; histone cluster 1, H2ah; histone cluster 1, H2ai;

histone cluster 1, H2ak; histone cluster 1, H2al; histone cluster 1, H2am

Homo sapiens

P59998 tubulin tyrosine ligase-like family, member 3; actin related protein 2/3

complex, subunit 4, 20kDa Homo sapiens

Q5DT20 hornerin Homo sapiens

Q9Y265 RuvB-like 1 (E. coli) Homo sapiens

P31948 stress-induced-phosphoprotein 1 Homo sapiens

P35659 DEK oncogene Homo sapiens

Q562Z4 actin, gamma 1 Homo sapiens

P14618 similar to Pyruvate kinase, isozymes M1/M2 (Pyruvate kinase muscle isozyme)

(Cytosolic thyroid hormone-binding protein) (CTHBP) (THBP1); pyruvate kinase,

muscle Homo sapiens

Q58F26 DEAH (Asp-Glu-Ala-His) box polypeptide 9 Homo sapiens

Q00765 receptor accessory protein 5 Homo sapiens

P23528 cofilin 1 (non-muscle) Homo sapiens

Q9Y2W1 thyroid hormone receptor associated protein 3 Homo sapiens

Q86TJ2 transcriptional adaptor 2 (ADA2 homolog, yeast)-beta Homo sapiens

P25445 Fas (TNF receptor superfamily, member 6) Homo sapiens

Q8NC51 SERPINE1 mRNA binding protein 1 Homo sapiens

P61769 beta-2-microglobulin Homo sapiens

P14317 hematopoietic cell-specific Lyn substrate 1 Homo sapiens

Q9H9Q2 COP9 constitutive photomorphogenic homolog subunit 7B (Arabidopsis)

Homo sapiens

Q6FI13 histone cluster 2, H2aa3; histone cluster 2, H2aa4 Homo sapiens

Q53HV1 ribosomal protein S4X pseudogene 6; ribosomal protein S4X pseudogene 13;

ribosomal protein S4, X-linked Homo sapiens

P39023 ribosomal protein L3; similar to 60S ribosomal protein L3 (L4)Homo sapiens

Q9UI08 Enah/Vasp-like Homo sapiens

P16070 CD44 molecule (Indian blood group) Homo sapiens

B2R549 ribosomal protein S13 pseudogene 8; ribosomal protein S13; ribosomal protein

S13 pseudogene 2 Homo sapiens

Q9BPX5 actin related protein 2/3 complex, subunit 5-like Homo sapiens

P11021 hypothetical gene supported by AF216292; NM_005347; heat shock 70kDa protein

5 (glucose-regulated protein, 78kDa) Homo sapiens

P60842 similar to eukaryotic translation initiation factor 4A; small nucleolar RNA,

H/ACA box 67; eukaryotic translation initiation factor 4A, isoform 1

Homo sapiens

O14672 ADAM metallopeptidase domain 10 Homo sapiens

O00299 chloride intracellular channel 1 Homo sapiens

Q13200 proteasome (prosome, macropain) 26S subunit, non-ATPase, 2Homo sapiens

Q99613 eukaryotic translation initiation factor 3, subunit C-likeHomo sapiens

Q9Y244 proteasome maturation protein Homo sapiens

P08962 CD63 molecule Homo sapiens

Q6GTX8 leukocyte-associated immunoglobulin-like receptor 1 Homo sapiens

P13164 interferon induced transmembrane protein 1 (9-27) Homo sapiens

Q96QU6 1-aminocyclopropane-1-carboxylate synthase homolog (Arabidopsis)(non-

functional) Homo sapiens

P49756 RNA binding motif protein 25 Homo sapiens

P23284 peptidylprolyl isomerase B (cyclophilin B) Homo sapiens

Q04637 eukaryotic translation initiation factor 4 gamma, 1 Homo sapiens

O15511 actin related protein 2/3 complex, subunit 5, 16kDa Homo sapiens

Q93096 protein tyrosine phosphatase type IVA, member 1 Homo sapiens

Q8N4C6 ninein (GSK3B interacting protein) Homo sapiens

P54728 RAD23b homolog (S. cerevisiae) Mus musculus

Q53R19 actin related protein 2/3 complex, subunit 2, 34kDa Homo sapiens

Q96EP5 DAZ associated protein 1 Homo sapiens

P13010 X-ray repair complementing defective repair in Chinese hamster cells 5

(double-strand-break rejoining) Homo sapiens

P04234 CD3d molecule, delta (CD3-TCR complex) Homo sapiens

P09327 villin 1 Homo sapiens

Q15424 scaffold attachment factor B Homo sapiens

A8K0N0 signal recognition particle 9-like 1; signal recognition particle 9kDa

Homo sapiens

P06127 CD5 molecule Homo sapiens

Q9UKV3 apoptotic chromatin condensation inducer 1 Homo sapiens

O14745 solute carrier family 9 (sodium/hydrogen exchanger), member 3 regulator 1

Homo sapiens

O75083 WD repeat domain 1 Homo sapiens

Q1KMD3 heterogeneous nuclear ribonucleoprotein U-like 2 Homo sapiens

P47756 capping protein (actin filament) muscle Z-line, beta Homo sapiens

P06729 CD2 molecule Homo sapiens

P30493 major histocompatibility complex, class I, C; major histocompatibility

complex, class I, B Homo sapiens

Q08499 phosphodiesterase 4D, cAMP-specific (phosphodiesterase E3 dunce homolog,

Drosophila) Homo sapiens

P04233 CD74 molecule, major histocompatibility complex, class II invariant chain

Homo sapiens

Q32MZ4 leucine rich repeat (in FLII) interacting protein 1 Homo sapiens

P00558 phosphoglycerate kinase 1 Homo sapiens

Q53FI1 lymphocyte cytosolic protein 1 (L-plastin) Homo sapiens

P51665 proteasome (prosome, macropain) 26S subunit, non-ATPase, 7Homo sapiens

P26038 moesin Homo sapiens

O75915 ADP-ribosylation-like factor 6 interacting protein 5 Homo sapiens

B2R6V5 ADP-ribosylation-like factor 6 interacting protein 5 Homo sapiens

Q02156 protein kinase C, epsilon Homo sapiens

P30203 CD6 molecule Homo sapiens

P28907 CD38 molecule Homo sapiens

Q0VD83 apolipoprotein B48 receptor Homo sapiens

P12544 granzyme A (granzyme 1, cytotoxic T-lymphocyte-associated serine esterase 3)

Homo sapiens

Q9NYF8 similar to Bcl-2-associated transcription factor 1 (Btf); BCL2-associated

transcription factor 1 Homo sapiens

O15127 secretory carrier membrane protein 2 Homo sapiens

Q15717 ELAV (embryonic lethal, abnormal vision, Drosophila)-like 1 (Hu antigen R)

Homo sapiens

O43169 cytochrome b5 type B (outer mitochondrial membrane) Homo sapiens

Q12874 splicing factor 3a, subunit 3, 60kDa Homo sapiens

Q96NB3 zinc finger protein 830 Homo sapiens

P68431 histone cluster 1, H3j; histone cluster 1, H3i; histone cluster 1, H3h;

histone cluster 1, H3g; histone cluster 1, H3f; histone cluster 1, H3e;

histone cluster 1, H3d; histone cluster 1, H3c; histone cluster 1, H3b;

histone cluster 1, H3a; histone cluster 1, H2ad; histone cluster 2, H3a;

histone cluster 2, H3c; histone cluster 2, H3d Homo sapiens

P50914 ribosomal protein L14 Homo sapiens

P41091 eukaryotic translation initiation factor 2, subunit 3 gamma, 52kDa

Homo sapiens

**Proteins detected only in exosomes from Jurkat cells**

UNIPROT_ACCESSION Name Species

Q8TF72 shroom family member 3 Homo sapiens

P10809 heat shock 60kDa protein 1 (chaperonin) pseudogene 5; heat shock 60kDa

protein 1 (chaperonin) pseudogene 6; heat shock 60kDa protein 1 (chaperonin)

pseudogene 1; heat shock 60kDa protein 1 (chaperonin) pseudogene 4; heat

shock 60kDa protein 1 (chaperonin) Homo sapiens

P63104 tyrosine 3-monooxygenase/tryptophan 5-monooxygenase activation protein, zeta

polypeptide Homo sapiens

Q6P595 signal recognition particle receptor, B subunit Homo sapiens

O75381 peroxisomal biogenesis factor 14 Homo sapiens

O95793 staufen, RNA binding protein, homolog 1 (Drosophila) Homo sapiens

P41208 centrin, EF-hand protein, 2 Homo sapiens

O00232 proteasome (prosome, macropain) 26S subunit, non-ATPase, 12Homo sapiens

O14776 transcription elongation regulator 1 Homo sapiens

Q15006 tetratricopeptide repeat domain 35 Homo sapiens

O94826 translocase of outer mitochondrial membrane 70 homolog A (S. cerevisiae)

Homo sapiens

O75531 similar to barrier-to-autointegration factor; barrier to autointegration

factor 1 Homo sapiens

P18077 ribosomal protein L35a Homo sapiens

Q8WTT2 nucleolar complex associated 3 homolog (S. cerevisiae) Homo sapiens

P09669 cytochrome c oxidase subunit VIc Homo sapiens

Q14152 eukaryotic translation initiation factor 3, subunit A Homo sapiens

Q5W0D7 vesicle transport through interaction with t-SNAREs homolog 1A (yeast)

Homo sapiens

Q9H444 chromatin modifying protein 4B Homo sapiens

P36543 ATPase, H+ transporting, lysosomal 31kDa, V1 subunit E1 Homo sapiens

Q86V81 THO complex 4 Homo sapiens

Q15413 ryanodine receptor 3 Homo sapiens

Q92896 golgi apparatus protein 1 Homo sapiens

Q96IX5 up-regulated during skeletal muscle growth 5 homolog (mouse)Homo sapiens

P63167 dynein, light chain, LC8-type 1 Homo sapiens

Q9BRV4 vesicle-associated membrane protein 3 (cellubrevin) Homo sapiens

P27449 ATPase, H+ transporting, lysosomal 16kDa, V0 subunit c Homo sapiens

Q5JTV8 torsin A interacting protein 1 Homo sapiens

O15258 RER1 retention in endoplasmic reticulum 1 homolog (S. cerevisiae) Homo

P33993 minichromosome maintenance complex component 7 Homo sapiens

Q8NBS9 thioredoxin domain containing 5 (endoplasmic reticulum); muted homolog

(mouse) Homo sapiens

Q00325 solute carrier family 25 (mitochondrial carrier; phosphate carrier), member 3

Homo sapiens

Q13601 KRR1, small subunit (SSU) processome component, homolog (yeast)Homo sapiens

Q9BWD2 splicing factor 3b, subunit 2, 145kDa Homo sapiens

Q8NC56 LEM domain containing 2 Homo sapiens

Q9BWJ5 splicing factor 3b, subunit 5, 10kDa Homo sapiens

Q9Y291 mitochondrial ribosomal protein S33 Homo sapiens

O60264 SWI/SNF related, matrix associated, actin dependent regulator of chromatin,

subfamily a, member 5 Homo sapiens

O95159 zinc finger protein-like 1 Homo sapiens

P06576 ATP synthase, H+ transporting, mitochondrial F1 complex, beta polypeptide

Homo sapiens

Q07666 KH domain containing, RNA binding, signal transduction associated 1

Homo sapiens

O95816 BCL2-associated athanogene 2 Homo sapiens

Q9HBB3 ribosomal protein L6 pseudogene 27; ribosomal protein L6 pseudogene 19;

ribosomal protein L6; ribosomal protein L6 pseudogene 10 Homo sapiens

Q9Y5Y5 peroxisomal biogenesis factor 16 Homo sapiens

P37268 farnesyl-diphosphate farnesyltransferase 1 Homo sapiens

O00231 proteasome (prosome, macropain) 26S subunit, non-ATPase, 11Homo sapiens

Q9C0H5 KIAA1688 protein Homo sapiens

O15226 NFKB repressing factor Homo sapiens

P16402 histone cluster 1, H1d Homo sapiens

O43809 nudix (nucleoside diphosphate linked moiety X)-type motif 21Homo sapiens

P62917 ribosomal protein L8; ribosomal protein L8 pseudogene 2 Homo sapiens

P37108 signal recognition particle 14kDa (homologous Alu RNA binding protein)

pseudogene 1; signal recognition particle 14kDa (homologous Alu RNA binding

protein) Homo sapiens

Q9BQE3 tubulin, alpha 1c Homo sapiens

Q9NW13 RNA binding motif protein 28 Homo sapiens

P49207 ribosomal protein L34 Homo sapiens

O95202 leucine zipper-EF-hand containing transmembrane protein 1 Homo sapiens

Q9UK45 LSM7 homolog, U6 small nuclear RNA associated (S. cerevisiae)Homo sapiens

P30040 endoplasmic reticulum protein 29 Homo sapiens

P42766 ribosomal protein L35; ribosomal protein L35 pseudogene 1; ribosomal protein

L35 pseudogene 2 Homo sapiens

P50402 emerin Homo sapiens

P26373 ribosomal protein L13 pseudogene 12; ribosomal protein L13Homo sapiens

P25788 proteasome (prosome, macropain) subunit, alpha type, 3 Homo sapiens

Q9Y277 voltage-dependent anion channel 3 Homo sapiens

Q01628 interferon induced transmembrane protein 3 (1-8U) Homo sapiens

Q9BSJ2 tubulin, gamma complex associated protein 2 Homo sapiens

O95876 chromosome 2 open reading frame 86 Homo sapiens

P62269 ribosomal protein S18 pseudogene 12; ribosomal protein S18 pseudogene 5;

ribosomal protein S18 Homo sapiens

Q5T7Y9 chromosome 1 open reading frame 77 Homo sapiens

Q9P0L0 VAMP (vesicle-associated membrane protein)-associated protein A, 33kDa

Homo sapiens

P13598 intercellular adhesion molecule 2 Homo sapiens

Q13765 nascent polypeptide-associated complex alpha subunit Homo sapiens

P19388 polymerase (RNA) II (DNA directed) polypeptide E, 25kDa Homo sapiens

P47914 ribosomal protein L29 pseudogene 9; ribosomal protein L29 pseudogene 12;

ribosomal protein L29 pseudogene 11; ribosomal protein L29; ribosomal

protein L29 pseudogene 26 Homo sapiens

P16989 cold shock domain protein A; cold shock domain protein A pseudogene 1

Homo sapiens

O94906 similar to U5 snRNP-associated 102 kDa protein (U5-102 kDa protein); PRP6

pre-mRNA processing factor 6 homolog (S. cerevisiae) Homo sapiens

P68366 tubulin, alpha 4a Homo sapiens

Q96FZ7 chromatin modifying protein 6 Homo sapiens

Q9NZ01 glycoprotein, synaptic 2 Homo sapiens

Q14697 glucosidase, alpha; neutral AB Homo sapiens

P63010 adaptor-related protein complex 2, beta 1 subunit Homo sapiens

P62424 ribosomal protein L7a pseudogene 70; ribosomal protein L7a; ribosomal

protein L7a pseudogene 30; ribosomal protein L7a pseudogene 66; ribosomal

protein L7a pseudogene 27; ribosomal protein L7a pseudogene 11; ribosomal

protein L7a pseudogene 62 Homo sapiens

Q7L1Q6 basic leucine zipper and W2 domains 1 pseudogene 1; basic leucine zipper and

W2 domains 1 like 1; basic leucine zipper and W2 domains 1Homo sapiens

P07602 prosaposin Homo sapiens

Q92841 DEAD (Asp-Glu-Ala-Asp) box polypeptide 17 Homo sapiens

P07737 profilin 1 Homo sapiens

P61289 proteasome (prosome, macropain) activator subunit 3 (PA28 gamma; Ki)

Homo sapiens

P62258 similar to 14-3-3 protein epsilon (14-3-3E) (Mitochondrial import

stimulation factor L subunit) (MSF L); tyrosine 3-monooxygenase/tryptophan 5-

monooxygenase activation protein, epsilon polypeptide Homo sapiens

P20292 arachidonate 5-lipoxygenase-activating protein Homo sapiens

Q16778 histone cluster 2, H2be Homo sapiens

Q5BKZ1 zinc finger protein 326 Homo sapiens

P22307 sterol carrier protein 2 Homo sapiens

Q9UMS4 PRP19/PSO4 pre-mRNA processing factor 19 homolog (S. cerevisiae)Homo sapiens

Q16718 NADH dehydrogenase (ubiquinone) 1 alpha subcomplex, 5, 13kDaHomo sapiens

O75964 ATP synthase, H+ transporting, mitochondrial F0 complex, subunit G

Homo sapiens

P28066 proteasome (prosome, macropain) subunit, alpha type, 5 Homo sapiens

Q15084 protein disulfide isomerase family A, member 6 Homo sapiens

Q5RKV6 exosome component 6 Homo sapiens

Q9UKR5 chromosome 14 open reading frame 1 Homo sapiens

P60866 ribosomal protein S20 Homo sapiens

P35268 ribosomal protein L22 pseudogene 11; ribosomal protein L22Homo sapiens

Q6NZ59 ATP synthase, H+ transporting, mitochondrial F0 complex, subunit F6

Homo sapiens

Q9H3Z4 DnaJ (Hsp40) homolog, subfamily C, member 5 Homo sapiens

Q9BRJ6 chromosome 7 open reading frame 50 Homo sapiens

O43823 A kinase (PRKA) anchor protein 8 Homo sapiens

Q9BZJ0 crooked neck pre-mRNA splicing factor-like 1 (Drosophila) Homo sapiens

Q9UEU0 vesicle transport through interaction with t-SNAREs homolog 1B (yeast)

Homo sapiens

Q9BRL6 splicing factor, arginine/serine-rich 2B Homo sapiens

O95292 VAMP (vesicle-associated membrane protein)-associated protein B and C

Homo sapiens

O75934 breast carcinoma amplified sequence 2 Homo sapiens

P62263 ribosomal protein S14 Homo sapiens

Q9P013 CWC15 spliceosome-associated protein homolog (S. cerevisiae)Homo sapiens

O60216 RAD21 homolog (S. pombe) Homo sapiens

Q92945 KH-type splicing regulatory protein Homo sapiens

Q86UE4 metadherin Homo sapiens

Q96AG4 leucine rich repeat containing 59 Homo sapiens

P82979 SAP domain containing ribonucleoprotein Homo sapiens

P30533 low density lipoprotein receptor-related protein associated protein 1

Homo sapiens

P48960 CD97 molecule Homo sapiens

Q9UG56 phosphatidylserine decarboxylase Homo sapiens

P24539 ATP synthase, H+ transporting, mitochondrial F0 complex, subunit B1

Homo sapiens

P52435 polymerase (RNA) II (DNA directed) polypeptide J, 13.3kDa Homo sapiens

Q9NQG5 regulation of nuclear pre-mRNA domain containing 1B Homo sapiens

Q9GZT3 chromosome 14 open reading frame 156 Homo sapiens

P05455 Sjogren syndrome antigen B (autoantigen La) Homo sapiens

Q96HY6 DDRGK domain containing 1 Homo sapiens

O43808 solute carrier family 25 (mitochondrial carrier; peroxisomal membrane protein, 34kDa), member 17 Homo sapiens

P84090 enhancer of rudimentary homolog (Drosophila) Homo sapiens

P55081 microfibrillar-associated protein 1 Homo sapiens

P0C0S8 histone cluster 1, H2ag; histone cluster 1, H2ah; histone cluster 1, H2ai;

histone cluster 1, H2ak; histone cluster 1, H2al; histone cluster 1, H2am

Homo sapiens

P08123 collagen, type I, alpha 2 Homo sapiens

P62316 small nuclear ribonucleoprotein D2 polypeptide 16.5kDa; similar to

hCG2040270 Homo sapiens

P56556 NADH dehydrogenase (ubiquinone) 1 alpha subcomplex, 6, 14kDaHomo sapiens

Q86YZ3 hornerin Homo sapiens

Q86Y82 syntaxin 12 Homo sapiens

Q8TF39 zinc finger protein 483 Homo sapiens

P31948 stress-induced-phosphoprotein 1 Homo sapiens

P14625 heat shock protein 90kDa beta (Grp94), member 1 Homo sapiens

Q08211 DEAH (Asp-Glu-Ala-His) box polypeptide 9 Homo sapiens

P49006 MARCKS-like 1 Homo sapiens

Q96NC0 zinc finger, matrin type 2 Homo sapiens

P61769 beta-2-microglobulin Homo sapiens

P51532 SWI/SNF related, matrix associated, actin dependent regulator of chromatin,

subfamily a, member 4 Homo sapiens

Q9Y6A9 signal peptidase complex subunit 1 homolog (S. cerevisiae)Homo sapiens

P25786 proteasome (prosome, macropain) subunit, alpha type, 1 Homo sapiens

P16401 histone cluster 1, H1b Homo sapiens

P21796 voltage-dependent anion channel 1; similar to voltage-dependent anion

channel 1 Homo sapiens

Q96MS3 glycosyltransferase 1 domain containing 1 Homo sapiens

Q9Y3U8 ribosomal protein L36; ribosomal protein L36 pseudogene 14Homo sapiens

Q9NRP0 oligosaccharyltransferase complex subunit; similar to DC2 protein

Homo sapiens

P62701 ribosomal protein S4X pseudogene 6; ribosomal protein S4X pseudogene 13;

ribosomal protein S4, X-linked Homo sapiens

Q8N5M9 jagunal homolog 1 (Drosophila) Homo sapiens

Q96QL0 ribosomal protein L3; similar to 60S ribosomal protein L3 (L4)Homo sapiens

Q8WY22 BRI3 binding protein Homo sapiens

Q7L2H7 eukaryotic translation initiation factor 3, subunit M Homo sapiens

Q9NUQ9 family with sequence similarity 49, member B Homo sapiens

Q9P2X0 dolichyl-phosphate mannosyltransferase polypeptide 3 Homo sapiens

Q96DV6 ribosomal protein S6 pseudogene 25; ribosomal protein S6; ribosomal protein

S6 pseudogene 1 Homo sapiens

Q14978 nucleolar and coiled-body phosphoprotein 1 Homo sapiens

P27348 tyrosine 3-monooxygenase/tryptophan 5-monooxygenase activation protein,

theta polypeptide Homo sapiens

P18669 phosphoglycerate mutase 1 (brain) Homo sapiens

P61254 ribosomal protein L26 pseudogene 33; ribosomal protein L26; ribosomal

protein L26 pseudogene 16; ribosomal protein L26 pseudogene 19; ribosomal

protein L26 pseudogene 6 Homo sapiens

P62854 ribosomal protein S26 pseudogene 38; ribosomal protein S26 pseudogene 39;

ribosomal protein S26 pseudogene 35; ribosomal protein S26 pseudogene 31;

ribosomal protein S26 pseudogene 20; ribosomal protein S26 pseudogene 54;

ribosomal protein S26 pseudogene 2; ribosomal protein S26 pseudogene 53;

ribosomal protein S26 pseudogene 25; ribosomal protein S26 pseudogene 50;

ribosomal protein S26 pseudogene 6; ribosomal protein S26 pseudogene 8;

ribosomal protein S26 Homo sapiens

Q71U36 tubulin, alpha 1a Homo sapiens

P55072 valosin-containing protein Homo sapiens

Q96MG7 necdin-like 2 Homo sapiens

P41223 BUD31 homolog (S. cerevisiae) Homo sapiens

Q6IB29 EBNA1 binding protein 2 Homo sapiens

Q15397 KIAA0020 Homo sapiens

Q5JRA6 melanoma inhibitory activity family, member 3 Homo sapiens

Q96A72 mago-nashi homolog B (Drosophila) Homo sapiens

Q9UF83 hypothetical LOC100271840 Homo sapiens

Q8IWV8 ubiquitin protein ligase E3 component n-recognin 2 Homo sapiens

P62249 ribosomal protein S16 pseudogene 1; ribosomal protein S16 pseudogene 10;

ribosomal protein S16 Homo sapiens

Q9BT78 COP9 constitutive photomorphogenic homolog subunit 4 (Arabidopsis) Homo Q96E43 oxysterol binding protein-like 8 Homo sapiens

P99999 cytochrome c, somatic Homo sapiens

P61604 heat shock 10kDa protein 1 (chaperonin 10) Homo sapiens

P16422 epithelial cell adhesion molecule Homo sapiens

P60468 Sec61 beta subunit Homo sapiens

Q8NA48 family with sequence similarity 71, member F1 Homo sapiens

P23396 ribosomal protein S3 pseudogene 3; ribosomal protein S3 Homo sapiens

Q8N5K1 CDGSH iron sulfur domain 2 Homo sapiens

Q92922 SWI/SNF related, matrix associated, actin dependent regulator of chromatin,

subfamily c, member 1 Homo sapiens

Q12996 cleavage stimulation factor, 3' pre-RNA, subunit 3, 77kDa Homo sapiens

Q14739 lamin B receptor Homo sapiens

Q8TDN6 brix domain containing 2 Homo sapiens

O14979 heterogeneous nuclear ribonucleoprotein D-like Homo sapiens

Q9H173 SIL1 homolog, endoplasmic reticulum chaperone (S. cerevisiae)Homo sapiens

Q9BTU6 phosphatidylinositol 4-kinase type 2 alpha Homo sapiens

Q9NX58 Ly1 antibody reactive homolog (mouse) Homo sapiens

Q9UNV9 similar to U5 snRNP-specific protein, 200 kDa; small nuclear

ribonucleoprotein 200kDa (U5) Homo sapiens

B2R4W8 ribosomal protein S15a pseudogene 17; ribosomal protein S15a pseudogene 19;

ribosomal protein S15a pseudogene 12; ribosomal protein S15a pseudogene 24;

ribosomal protein S15a pseudogene 11; ribosomal protein S15aHomo sapiens

P35613 basigin (Ok blood group) Homo sapiens

Q15067 acyl-Coenzyme A oxidase 1, palmitoyl Homo sapiens

Q15029 elongation factor Tu GTP binding domain containing 2 Homo sapiens

O95183 vesicle-associated membrane protein 5 (myobrevin) Homo sapiens

P62312 LSM6 homolog, U6 small nuclear RNA associated (S. cerevisiae)Homo sapiens

P42166 thymopoietin Homo sapiens

P30825 solute carrier family 7 (cationic amino acid transporter, y+ system), member

1 Homo sapiens

Q15822 cholinergic receptor, nicotinic, alpha 2 (neuronal) Homo sapiens

O95298 NADH dehydrogenase (ubiquinone) 1, subcomplex unknown, 2, 14.5kDa Homo

Q99459 CDC5 cell division cycle 5-like (S. pombe) Homo sapiens

P62314 small nuclear ribonucleoprotein D1 polypeptide 16kDa; hypothetical protein

LOC100129492 Homo sapiens

P62306 small nuclear ribonucleoprotein polypeptide F Homo sapiens

O43395 PRP3 pre-mRNA processing factor 3 homolog (S. cerevisiae) Homo sapiens

Q96G25 mediator complex subunit 8 Homo sapiens

P02452 collagen, type I, alpha 1 Homo sapiens

P49458 signal recognition particle 9-like 1; signal recognition particle 9kDa

Homo sapiens

P61326 mago-nashi homolog, proliferation-associated (Drosophila) Homo sapiens

Q9NZ45 CDGSH iron sulfur domain 1 Homo sapiens

P36578 ribosomal protein L4; ribosomal protein L4 pseudogene 5; ribosomal protein

L4 pseudogene 4 Homo sapiens

P35232 prohibitin Homo sapiens

P53801 pituitary tumor-transforming 1 interacting protein Homo sapiens

Q15652 jumonji domain containing 1C Homo sapiens

P62318 small nuclear ribonucleoprotein D3 polypeptide 18kDa Homo sapiens

P04350 tubulin, beta 4 Homo sapiens

P45880 voltage-dependent anion channel 2 Homo sapiens

Q5U0B3 proteasome (prosome, macropain) 26S subunit, non-ATPase, 8Homo sapiens

Q96A26 family with sequence similarity 162, member A Homo sapiens

O95983 methyl-CpG binding domain protein 3 Homo sapiens

Q6I9Y2 THO complex 7 homolog (Drosophila) Homo sapiens

P67809 Y box binding protein 1 Homo sapiens

Q6IB68 c-myc binding protein Homo sapiens

Q8WXI9 GATA zinc finger domain containing 2B Homo sapiens

P53999 SUB1 homolog (S. cerevisiae) Homo sapiens

Q13033 striatin, calmodulin binding protein 3 Homo sapiens

Q9NQX3 gephyrin Homo sapiens

Q8NDV3 structural maintenance of chromosomes 1B Homo sapiens

Q9P0S2 COX16 cytochrome c oxidase assembly homolog (S. cerevisiae)Homo sapiens

Q9Y3B4 splicing factor 3B, 14 kDa subunit Homo sapiens

Q70UQ0 IKK interacting protein Homo sapiens

Q13813 spectrin, alpha, non-erythrocytic 1 (alpha-fodrin) Homo sapiens

Q8IWZ8 splicing factor 4 Homo sapiens

P51398 death associated protein 3 Homo sapiens

P51812 ribosomal protein S6 kinase, 90kDa, polypeptide 3 Homo sapiens

P51991 heterogeneous nuclear ribonucleoprotein A3 Homo sapiens

P62805 histone cluster 1, H4l; histone cluster 1, H4k; histone cluster 4, H4;

histone cluster 1, H4h; histone cluster 1, H4j; histone cluster 1, H4i;

histone cluster 1, H4d; histone cluster 1, H4c; histone cluster 1, H4f;

histone cluster 1, H4e; histone cluster 1, H4b; histone cluster 1, H4a;

histone cluster 2, H4a; histone cluster 2, H4b Homo sapiens

P33947 KDEL (Lys-Asp-Glu-Leu) endoplasmic reticulum protein retention receptor 2

Homo sapiens

P20963 CD247 molecule Homo sapiens

O95926 SYF2 homolog, RNA splicing factor (S. cerevisiae) Homo sapiens

P16104 H2A histone family, member X Homo sapiens

O95249 golgi SNAP receptor complex member 1 Homo sapiens

Q53XN6 ELAV (embryonic lethal, abnormal vision, Drosophila)-like 1 (Hu antigen R)

Homo sapiens

Q9BQG0 MYB binding protein (P160) 1a Homo sapiens

P04844 ribophorin II Homo sapiens

Q86W42 THO complex 6 homolog (Drosophila) Homo sapiens

P68431 histone cluster 1, H3j; histone cluster 1, H3i; histone cluster 1, H3h;

histone cluster 1, H3g; histone cluster 1, H3f; histone cluster 1, H3e;

histone cluster 1, H3d; histone cluster 1, H3c; histone cluster 1, H3b;

histone cluster 1, H3a; histone cluster 1, H2ad; histone cluster 2, H3a;

histone cluster 2, H3c; histone cluster 2, H3d Homo sapiens

P54920 N-ethylmaleimide-sensitive factor attachment protein, alphaHomo sapiens

Q01844 similar to Ewing sarcoma breakpoint region 1; Ewing sarcoma breakpoint

region 1 Homo sapiens
